# Developing a new host-vector system for *Deinococcus grandis*

**DOI:** 10.3389/fmicb.2024.1387296

**Published:** 2024-05-28

**Authors:** Miyabi Sakai, Taichi Shimosaka, Kosuke Katsumata, Masafumi Yohda, Issay Narumi

**Affiliations:** ^1^Department of Biotechnology and Life Science, Faculty of Engineering, Tokyo University of Agriculture and Technology, Koganei, Tokyo, Japan; ^2^Department of Life Sciences, Faculty of Life Sciences, Toyo University, Asaka, Japan; ^3^Graduate School of Life Sciences, Toyo University, Asaka, Japan

**Keywords:** *Deinococcus grandis*, host-vector system, plasmid, shuttle vector, gene expression, copy number

## Abstract

*Deinococcus* spp. are known for their radiation resistance, toxic compound removal, and production of valuable substances. Therefore, developing gene expression systems for *Deinococcus* spp. is crucial in advancing genetic engineering applications. To date, plasmid vectors that express foreign genes in *D. radiodurans* and *D. geothermalis* have been limited to plasmid pI3 and its derivatives. In contrast, plasmid vectors that express foreign genes in *D. grandis* include plasmid pZT23 and its derivatives. In this study, we developed a new system for the stable introduction and retention of expression plasmids for *D. grandis*. Two cryptic plasmids were removed from the wild-type strain to generate the TY3 strain. We then constructed a shuttle vector plasmid, pGRC5, containing the replication initiation region of the smallest cryptic plasmid, pDEGR-3, replication initiation region of the *E. coli* vector, pACYC184, and an antibiotic resistance gene. We introduced pGRC5, pZT23-derived plasmid pZT29H, and pI3-derived plasmid pRADN8 into strain TY3, and found their coexistence in *D. grandis* cells. The quantitative PCR assay results found that pGRC5, pZT29H, and pRADN8 had relative copy numbers of 11, 26, and 5 per genome, respectively. Furthermore, we developed a new plasmid in which the luciferase gene was controlled by the promoter region, which contained radiation-desiccation response operator sequences for *D. grandis* DdrO, a stress response regulon repressor in *D. grandis*, hence inducing gene expression via ultraviolet-C light irradiation. These plasmids are expected to facilitate the removal and production of toxic and valuable substances, in *D. grandis*, respectively, particularly of those involving multiple genes.

## 1 Introduction

When genes capable of removing persistent or harmful substances or genes involved in the intracellular uptake of chemical elements are introduced or expressed in radioresistant bacteria using genetic engineering methods, persistent and harmful substances contained in radionuclide-contaminated waste can be removed. Therefore, the genetic engineering of radioresistant bacteria is helpful as a bioremediation technology for radionuclide removal (Gabani and Singh, 2013; Gerber et al., 2015; Li et al., 2021).

*Deinococcus* spp. are the most studied radioresistant bacteria and can be genetically engineered; *Deinococcus radiodurans* is the most researched species. It has a multiploid genome (4–10 copies per cell) and a high natural transformation potential (Moseley and Setlow, 1968; Hansen, 1978; Harsojo et al., 1981; Ithurbide et al., 2020). The radiation resistance of *D. radiodurans* is 250 times that of *Escherichia coli* and approximately 1,000 times that of most vertebrates. This ability to withstand high radiation levels is primarily attributed to its adequate protein protection by the antioxidant defenses and exceptionally high DNA repair capacity (Cox and Battista, 2005; Ishino and Narumi, 2015; Lim et al., 2019; Daly, 2023; Sadowska-Bartosz and Bartosz, 2023).

Some examples of bioremediation techniques using *D. radiodurans* include expression of a toluene dioxygenase gene from *Pseudomonas putida* in *D. radiodurans* to convert persistent toluene into an easily degradable substance (Lange et al., 1998). A divalent mercury ion reduction gene from *E. coli* was expressed in *D. radiodurans* to reduce divalent mercury ions to the less toxic and volatile metallic mercury (Brim et al., 2000). Using *D. radiodurans* as a host, a nonspecific acid phosphatase gene from *Salmonella enterica* Typhi or an alkaline phosphatase gene from *Sphingomonas* sp. was expressed on a plasmid to precipitate uranium from a uranyl nitrate solution (Appukuttan et al., 2006). An endoglucanase gene from *Bacillus pumilus* was expressed on plasmids to contribute to the bioremediation of cellulosic waste in radioactive environments (Telang et al., 2014). A synthetic gene that encoded a phytochelatin analog or a metallothionein gene from *Synechococcus* sp. was expressed on a plasmid to improve the cadmium accumulation capacity (Chaturvedi and Archana, 2014). A nickel/cobalt transporter gene from *Rhodopseudomonas palustris* or *Novosphingobium aromaticivorans* was expressed on plasmids to improve the efficiency of radioactive cobalt elimination from radioactively contaminated water (Gogada et al., 2015). Another example involves the expression of a divalent mercury ion reduction gene derived from *E. coli* a plasmid using *Deinococcus geothermalis* as a host; divalent mercury ions were reduced to less toxic and volatile metallic mercury (Brim et al., 2003).

Plasmid vectors for foreign gene expression using *D. radiodurans* or *D. geothermalis* as hosts were limited to *D. radiodurans-E. coli* shuttle vector pI3 and its derived plasmids, which were developed using replicons of plasmid pUE10 from the *D. radiodurans* strain Sark and an *E. coli* vector (Masters and Minton, 1992; Meima and Lidstrom, 2000). It has been reported that pI3-based plasmids are also replicate in *Deinococcus deserti* (Dulermo et al., 2009).

*D. grandis* is a radioresistant bacterium isolated from carp intestines in Hino, Japan. A host vector system has been developed for this bacterium (Satoh et al., 2009). In the *D. grandis-E. coli* shuttle vector pZT23 and its derived plasmids, the replicon of the cryptic plasmid pUE30 from *Deinococcus radiopugnans* functions in *D. grandis*. To investigate a specific gene function in *D. grandis* using the developed host-vector system, a plasmid complementation study was conducted using a host strain of *D. grandis* with the deletion of the *rodZ* gene (Morita et al., 2019). However, examples of plasmids with replicons other than pUE30 replicating in *D. grandis* have not been reported.

Unlike *D. radiodurans*, *D. grandis* has the rod-shape related genes, *mreBC* and *rodAZ*, and exhibits a rod-shaped morphology (Morita and Nishida, 2018). MreB, the bacterial ancestor of eukaryotic actin, functions as a scaffold for the assembly of cell wall synthesis machinery (van den Ent et al., 2001; Pande et al., 2022). MreC, a membrane-spanning protein with a single transmembrane domain, is required for correct localization of the MreB filament (Kruse et al., 2005). RodA is an integral membrane protein that is involved in the translocation of the lipid II peptidoglycan precursors across the cytoplasmic membrane (den Blaauwen et al., 2008; Sieger et al., 2013). RodZ is a transmembrane protein that directly interacts with the bacterial tubulin homolog FtsZ, and recruits MreB to the divisome (Ago and Shiomi, 2019). In general, rod-shaped microbes have a larger surface area-to-volume ratio than cocci (Harris and Theriot, 2018), giving them an advantage as hosts for bioremediation. *D. grandis* is also known to form enlarged spheroplasts with large periplasmic spaces under certain culture conditions, and these enlarged spheroplasts fuse to form giant cells with multiple cytoplasms (Nishino et al., 2018, 2019;Morita et al., 2019; Nishino and Nishida, 2019). The large periplasmic spaces have attractive potential as reaction sites for bioremediation and production of valuable substances. This peculiar property motivated the development of a new host-vector system for *D. grandis*.

When DNA regions with the same sequence are present on multiple plasmids, homologous recombination reactions occur between plasmids at a high frequency because of the action of relevant repair proteins, complicating the stable maintenance of various plasmids within bacteria (Masters et al., 1991; Daly et al., 1994). In addition, the DNA fragment size that can be stably incorporated into a plasmid vector is generally limited to approximately 10 kb (Leonardo and Sedivy, 1990; Siguret et al., 1994). Hence, expression plasmids that incorporate large DNA fragments are considered unstable in host cells. Therefore, when transforming a host with multiple genes, it is desirable to use multiple plasmid vectors. However, the repertoire of known plasmid vectors available for *D. grandis* was limited (Satoh et al., 2009).

## 2 Materials and methods

### 2.1 Bacterial strains and growth conditions

*Deinococcus grandis* ATCC43672^T^ and *D. radiodurans* ATCC13939^T^ were purchased from the American Type Culture Collection. *E. coli* strain JM109 was purchased from Takara Bio. Inc. (Shiga, Japan) (Table 1). *D. grandis* and *D. radiodurans* were grown in tryptone glucose yeast (TGY) broth containing 0.5% Bacto Tryptone (BD, NJ, United States), 0.1% glucose, 0.3% Bacto Yeast Extract (BD), or on TGY agar supplemented with 1.5% Bacto Agar (BD) at 30°C, unless otherwise mentioned. *E. coli* was grown in LB broth-Lennox or LB Agar-Lennox (BD) at 37°C. The following antibiotics were added as needed: for *D. grandis*, chloramphenicol (3 μg/mL), hygromycin B (50 μg/mL), streptomycin (2 μg/mL); for *E. coli*, chloramphenicol (15 μg/mL), hygromycin B (100 μg/mL), streptomycin (17.5 μg/mL) or spectinomycin (100 μg/mL).

**Table 1:** Strains and plasmids used in this study.

Designation	Relevant description	Source or references
*D. grandis*		
ATCC43672	Wild type	ATCC
TY1	As wild type but pDEGR-PL^−^	This study
TY3	As wild type but pDEGR-PL^−^ pDEGR-3^−^	This study
*D. radiodurans*		
ATCC13939	Wild type	ATCC
***E. coli***		
JM109	Host for recombinant plasmids	Takara Bio
*Plasmids*		
pDEGR-PL	*D. grandis* cryptic plasmid; 91,291 bp	Satoh et al. (2016)
pDEGR-3	*D. grandis* cryptic plasmid; 8,055 bp	Shibai et al. (2019)
pKatAAD2	pUC19-based vector containing *D. radiodurans* catalase promoter (*kat*p*) and *aad* (KatAAD cassette); 2,241 bp	Satoh et al. (2006)
pKatAAD2L	pACYC184-based vector containing KatAAD cassette; 1,900 bp	This study
pKatHPH4	pUC19-based vector containing KatHPH cassette, hygromycin resistance version of KatAAD cassette; 2,492 bp	Satoh et al. (2006)
pZT29	*E. coli*-*D. grandis* shuttle vector carrying pUC19 and pUE10 replicons with KatCAT cassette; 4,318 bp	Satoh et al. (2009)
pZT29H	*E. coli*-*D. grandis* shuttle vector carrying pUC19 and pUE10 replicons with KatHPH cassette; 4,601 bp	This study
pGRC5	*E. coli*-*D. grandis* shuttle vector carrying p15A and pDEGR-3 replicons with KatAAD cassette; 3,908 bp	This study
pRADN1	*E. coli*-*D. radiodurans* shuttle vector carrying pMTL23 and pUE30 replicons with *bla* and *cat*; 6,809 bp	Ohba et al. (2005)
pKatCAT5	pUC19-based vector containing KatCAT cassette, chloramphenicol version of KatAAD cassette; 2,209 bp	Satoh et al. (2009)
pRADN7	*E. coli*-*D. radiodurans* shuttle vector carrying pUC19 and pUE30 replicons with KatCAT cassette; 5,333 bp	This study
pGBM5	pSC101-based *E. coli* cloning vector carrying *aad*; 4,447 bp	National Institute of Genetics, Japan
pRADN8	*E. coli*-*D. grandis* shuttle vector carrying pSC101 and pUE30 replicons with KatCAT cassette; 6,046 bp	This study
pNL1.1[Nluc]	*E. coli* vector carrying deep sea shrimp luciferase gene (*Nluc*); 3,110 bp	Promega
pRNKAAD	pKatAAD2 containing *Nluc* controlled by *D. radiodurans ddrO* promoter (*ddrO*p*-Nluc*). Used to construct plasmids pΔ500008LA and pRDR-Nluc. 3,025 bp	This study
pΔ500008LA	pRNKAAD containing the upstream and downstream regions of DEIGR_500008. Used to generate strain TY1Nluc. 5,905 bp	This study
pDEGR-3Δ8LA	pDEGR-3 but DEIGR_500008 was replaced by *ddrO*p*-Nluc* and KatAAD cassette; 8,961 bp	This study
pGEM-T	*E. coli* vector for TA cloning; 3,000 bp	Promega
pDgra-dnaA	pGEM-T containing *D. grandis dnaA.* Used to produce standard curve for quantitative PCR. 3,101 bp	This study
pDrad-dnaA	pGEM-T containing *D. radiodurans dnaA.* Used to produce standard curve for quantitative PCR. 3,118 bp	This study
pZT90	pZT29 containing *D. radiodurans groES* minimal promoter (*groE*p). Used to construct plasmid pGNKAAD. 4,459 bp	Satoh et al. (2009)
pGNKAAD	pKatAAD2 containing *groE*p and *Nluc* (*groE*p*-Nluc* cassette). Used to construct plasmids pGRC5GN, pZT29HGN, and pRADN8GN. 2,914 bp	This study
pGRC5GN	pGRC5 containing *groE*p*-Nluc* cassette; 4,545 bp	This study
pZT29HGN	pZT29H containing *groE*p*-Nluc* cassette; 5,250 bp	This study
pRADN8GN	pRADN8 containing *groE*p*-Nluc* cassette; 6,683 bp	This study
pRDR-Nluc	pGRC5 containing *ddrO*p*-Nluc* cassette; 4,688 bp	This study
pZTGL93	pZT90 containing *groE*p and firefly luciferase gene (*luc*+) (*groE*p-*luc* + cassette). Used to construct plasmid pRDR-NlucD. 6,107 bp	Satoh et al. (2009)
pRDR-NlucD	pRDR-Nluc containing *groE*p-*luc* + cassette; 6,479 bp	This study

*katp: the − 35 promoter sequence TGGACA was changed to TTGACA to allow for streptomycin/spectinomycin selection of the plasmids in *E. coli*.

### 2.2 Polymerase chain reaction

Genomic DNA was extracted from *D. grandis* and *D. radiodurans* using a FastDNA SPIN Kit (MP Biomedicals, CA, United States) and a FastPrep 24 Instrument Version 4 (MP Biomedicals). PCR was performed using Tks Gflex DNA polymerase (Takara Bio. Inc.) or AmpliTaq Gold 360 DNA polymerase (Thermo Fisher Scientific, MA, United States). PCR was performed as per manufacturer’s instructions. Oligonucleotide primers used in this study are listed in Supplementary Table 1. PCR products were purified using a Gel/PCR Extraction Kit (Nippon Genetics Co., Ltd., Tokyo, Japan).

### 2.3 Construction of plasmid pΔ500008LA

A 263-bp DNA fragment containing the promoter for the DNA damage response repressor-encoding *ddrO* gene was PCR-amplified using *D. grandis* genomic DNA as a template and treated with *Kpn*I and *Nco*I. In addition, using the pNL1.1[Nluc] vector (Promega, WI, United States) as a template, a 564-bp DNA fragment of the luciferase gene from deep sea shrimp *Oplophorus gracilirostris* was PCR-amplified and treated with *Nco*I and *Xho*I. These were ligated to the *Kpn*I-*Xho*I site of pKatAAD2 to construct plasmid pRNKAAD. Fragments upstream and downstream of the DEIGR_500008 gene were PCR-amplified using *D. grandis* genomic DNA as a template (769 bp and 770 bp, respectively); they were mixed and treated with the restriction enzymes *Eco*RI, *Kpn*I, *Pst*I, and *Hin*dIII. In addition, a PCR-amplified fragment of 3,025 bp containing the *Nluc* and streptomycin resistance gene (*aad*), using the plasmid pRNKAAD as a template, was treated with *Kpn*I and *Pst*I. These three treated fragments were ligated to the *Eco*RI-*Hin*dIII site of pUC19 to construct the plasmid pΔ500008LA (Supplementary Figure 1). Plasmids used in this study are listed in Table 1.

### 2.4 Generation of *Deinococcus grandis* strains TY1 and TY3

To generate *D. grandis* strain TY1, wild-type strain was incubated at 40°C for 48 h with shaking and then spread on TGY agar. Ten colonies formed after incubation at 30°C for 48 h were randomly selected, inoculated into TGY broth, and incubated at 30°C for 24 h.

To generate *D. grandis* strain TY3, wild-type strain was transformed with PCR products that were amplified with primers pKat-FP2 and pKat-RP (Supplementary Table 1) using pΔ500008LA as a template to generate strain TY1Nluc. The strain was cultured in TGY broth supplemented with 5 μg/mL rifampicin at 40°C for 48 h with shaking, diluted 100-fold, inoculated in the same medium, and cultured at 40°C for 72 h with shaking. The culture was then spread on TGY agar and incubated at 30°C for 48 h. Luciferase-nonproductive and streptomycin-sensitive colonies were selected from TGY agar, inoculated into TGY broth, and incubated at 30°C for 24 h.

### 2.5 Shuttle vector plasmid construction

Using the *E. coli* vector pACYC184 (Chang and Cohen, 1978) as a template, a 933-bp DNA fragment containing the p15A replicon was PCR-amplified and treated with *Sph*I and *Kpn*I. Plasmid pKatAAD2 (Satoh et al., 2006) was also treated with *Sph*I and *Kpn*I to obtain a 981-bp DNA fragment. These DNA fragments were ligated to yield the plasmid pKatAAD2L. A 2,022-bp DNA fragment containing the replicon of plasmid pDEGR-3 of *D. grandis* was then PCR-amplified, treated with *Kpn*I, and ligated to the *Kpn*I site of pKatAAD2 to construct plasmid pGRC5 (Supplementary Figure 2).

A 2,109-bp DNA fragment of the shuttle vector pZT29 (Satoh et al., 2009), which contained the replicon of *D. radiopugnans* plasmid pUE30 and that of *E. coli* vector pUC19 treated with *Kpn*I, was ligated to a 2,492-bp DNA fragment of plasmid pKatHPH4 (Satoh et al., 2006) treated with *Kpn*I, to construct plasmid pZT29H. In this plasmid, the chloramphenicol resistance gene, a marker gene of pZT29, was replaced with a hygromycin resistance gene (Supplementary Figure 3).

Using plasmid pRADN1 (Ohba et al., 2005) as a template, a 3,164-bp DNA fragment containing the pUE10 replicon was PCR-amplified and treated with *Xho*I and *Eco*RV. The plasmid pKatCAT5 (Satoh et al., 2009) was treated with *Xho*I and *Eco*RV. These fragments were ligated to construct a plasmid pRADN7. Using the *E. coli* vector pGBM5 as a template, a 1,997-bp fragment containing the replicon was then PCR-amplified and treated with *Xho*I and *Hin*dIII. Plasmid pRADN7 was treated with *Xho*I and *Hin*dIII to obtain a 4,063-bp DNA fragment. These DNA fragments were ligated to construct a plasmid pRADN8 (Supplementary Figure 4).

### 2.6 Plasmid transformation and extraction from transformants

*Escherichia coli* cells were transformed with plasmids using an ECM 399 Electroporation System (BTX, MA, United States). *D. grandis* was transformed with plasmids using the calcium chloride method based on previous studies. Streptomycin was used as a selection marker for the pGRC5-transformants, chloramphenicol for the pRADN8-transformants, and hygromycin B for the pZT29H-transformants.

Plasmids from *the E. coli* transformants were extracted as per the standard protocol of the FastGene Plasmid Mini Kit (Nippon Genetics). Transformant cultures of *D. grandis* (20 mL) were collected via centrifugation and resuspended in buffer mP1 (Nippon Genetics) containing 10 mg/mL lysozyme. After incubating the suspension at 37°C for 30 min, plasmid DNA was extracted using the FastGene Plasmid Mini Kit, as per manufacturer’s instructions.

### 2.7 Determination of plasmid copy number

Quantitative PCR (qPCR) was performed to determine the copy number of shuttle vectors in *D. grandis* transformants essentially as described (Lee et al., 2006). Total DNA was extracted using the SPINeasy DNA Kit for Microbiome (MP Biomedicals). *dnaA* was selected as a target gene on the *D. grandis* chromosome. A 101-bp *dnaA* fragment was PCR-amplified and ligated with TA-cloning vector pGEM-T (Promega) to yield pDgra-dnaA. This plasmid was used to produce standard curves for the *dnaA* target. pDEGR-3 *rep*, pUE10 *rep*, and pUE30 *rep* were selected as targets for pGRC5, pRADN8, and pZT29H, respectively. Plasmid copy number was calculated as the ratio of quantified *rep* to *dnaA*. Oligonucleotide primers for qPCR analysis are listed in Supplementary Table 1. qPCR was carried out using a QuantStudio 1 Real-time PCR System (ThermoFisher Scientific) with PowerTrack SYBR Green Master Mix (ThermoFisher Scientific) according to the manufacturer’s instructions. The final reaction volume was 20 μL. Standard cycling mode was taken as thermal protocol. A tenfold serial dilution series of plasmids containing *dnaA* or *rep* was prepared ranging from 1 × 10^−5^ to 1 × 10^−9^ ng/reaction and standard curves displaying the cycle threshold parameter (Ct) values plotted against the log of the initial DNA concentration were generated. Plasmid copies were calculated based on the amplicon size of the target genes and the weight of 1 bp (1.095 × 10^−12^ ng). The relative copy numbers of *dnaA* and *rep* genes in samples were determined from qPCR analysis using extracted total DNA with reference to the standard curves.

Plasmid copy number of pRAND8 in *D. radiodurans* was also determined using qPCR. In this case, a 118-bp *D. radiodurans dnaA* fragment was PCR-amplified and ligated with TA-cloning vector pGEM-T to yield pDrad-dnaA, and this plasmid was used to produce standard curves for the *dnaA* target.

### 2.8 Evaluation of plasmid stability

Using plasmid pZT90 (Satoh et al., 2009) as a template, a 152-bp fragment containing the *D. radiodurans groES* minimal promoter region was PCR-amplified and treated with *Kpn*I and *Nco*I. This DNA fragment was inserted into the *Kpn*I-*Nco*I site of pRNKAAD to construct pGNKAAD (Supplementary Figure 5). Next, three plasmids were constructed to evaluate the plasmid stability in the transformants without selection pressure. (1) A 669-bp fragment (*groE*p*-Nluc* cassette) containing the *D. radiodurans groES* minimal promoter and deep sea shrimp luciferase gene amplified using pGNKAAD as a template was treated with *Bam*HI and *Pst*I and inserted into the *Sal*I-HindIII site of the shuttle vector pGRC5 to yield the luciferase gene expression plasmid pGRC5GN. (2) The PCR-amplified *groE*p*-Nluc* cassette (667 bp) using pGNKAAD as a template was treated with *Sal*I and *Hin*dIII and inserted into the *Sal*I-*Hin*dIII site of shuttle vector pRADN8 to yield pRADN8GN. (3) The PCR-amplified *groE*p*-Nluc* cassette (669 bp), using pGNKAAD as a template, was treated with *Bam*HI and *Xba*I and inserted into the *Bam*HI-*Xba*I site of shuttle vector pZT29H to yield pZT29HGN (Supplementary Figure 6).

Evaluation of plasmid stability was performed as described previously (Satoh et al., 2009). *D. grandis* transformants (strain TY3 carrying pGRC5GN, pRADN8, or pZT29HGN) were cultivated for 24 h in TGY broth supplemented with antibiotics. Cultures were diluted 4,096-fold in TGY broth without antibiotic addition and cultivated for 24 h. Following cell division through 12 generations, cultures were again diluted 4,096-fold in TGY broth. This procedure was repeated until cell division reached 48 generations. At each dilution step, ten milliliters of the culture were transferred to flat-bottomed microplates with 5 mm diameter, and luciferase activity was determined using a Nano-Glo Luciferase Assay System (Promega). Chemiluminescence signals were captured using a LumiCube Chemiluminescence Imaging System (Liponics, Tokyo, Japan) with the Digital Photo Professional 4 software (ver. 4.10.40, CANON) for 5 s at the optimal ISO sensitivity and signal intensities were measured using the JustTLC software (ver. 4.6.3, Sweday, Stockholm, Sweden). Relative chemiluminescence intensity was calculated by setting the chemiluminescence intensity at the 0 generation to 1.

### 2.9 Induction of gene expression via UV irradiation

A 263-bp promoter fragment for the DNA damage response repressor-encoding *ddrO* gene was PCR-amplified using *D. grandis* genomic DNA and treated with *Xho*I and *Nco*I. In addition, a 564-bp fragment of the deep sea shrimp luciferase gene was PCR-amplified using the pNL1.1[Nluc] vector (Promega) and treated with *Nco*I and *Hin*dIII. These were ligated to the *Xho*I-*Hin*dIII site of pGRC5 to construct the plasmid pRDR-Nluc. A PCR product containing the *D. radiodurans groES* minimal promoter and its downstream region containing the firefly luciferase gene *luc* + (*groE*p*-luc*+) cloned into pZTGL93 (Satoh et al., 2009) was treated with *Eco*RI and *Sac*I, and then inserted into the *Eco*RI-*Sac*I site of pRDR-Nluc to yield the dual-luciferase gene expression plasmid pRDR-NlucD (Supplementary Figure 7). This plasmid was used to transform the *D. grandis* strain TY3 carrying plasmid pZT29H.

The culture of *D. grandis* transformants (strain TY3 carrying pRDR-NlucD and pZT29H; 1 mL) grown in TGY broth supplemented with streptomycin and hygromycin for 18 h was washed with 1 mL of 10 mM sodium phosphate buffer (pH 7.0) and resuspended in 0.4 mL of the same buffer. The suspensions were transferred to a 35-mm diameter polystyrene dish (Model 3,000–035, AGC Techno Glass Co., Ltd., Shizuoka, Japan) and irradiated at room temperature at constant UV irradiation of 4.2 J/m^2^/s using four UV lamps. To 0.1 mL of the irradiated suspension, a 25-fold volume of TGY broth supplemented with streptomycin and hygromycin was added and incubated at 30°C for 4.5 h. Every 30 min, 10 μL of the culture was withdrawn and luciferase activity was measured using the Nano-Glo Dual-Luciferase Reporter Assay System (Promega), as described in Section 2.8. Relative reporter activity was calculated as the ratio of chemiluminescence from the experimental reporter (*Nluc*) to that from the control reporter (*luc*+). Normalized relative reporter activity was calculated via setting the relative reporter activity at 0 min for each dose to 1.

### 2.10 Statistical analysis

The data of the plasmid copy number estimation in Section 2.7 was reported as an average of four qPCR assays. The data of the relative chemiluminescence intensity in Section 2.8 was presented as mean ± standard error (*n* = 9). The data of the normalized relative reporter activity in Section 2.9 was presented as mean ± standard error (*n* = 3).

## 3 Results

### 3.1 Generation of *Deinococcus grandis* strain TY1

Various methods have been developed to delete plasmids from microorganisms (Trevors, 1986; Spengler et al., 2006). In this study, we attempted to culture *D. grandis* at 40°C, the upper permissive temperature limit for *D. grandis*, as describes in Section 2.4. The presence of the plasmid pDEGR-3 in bacteria was confirmed using agarose gel electrophoresis. The plasmid was detected in all isolates regardless of incubation at 40°C (Supplementary Figure 8).

**Figure 1:**
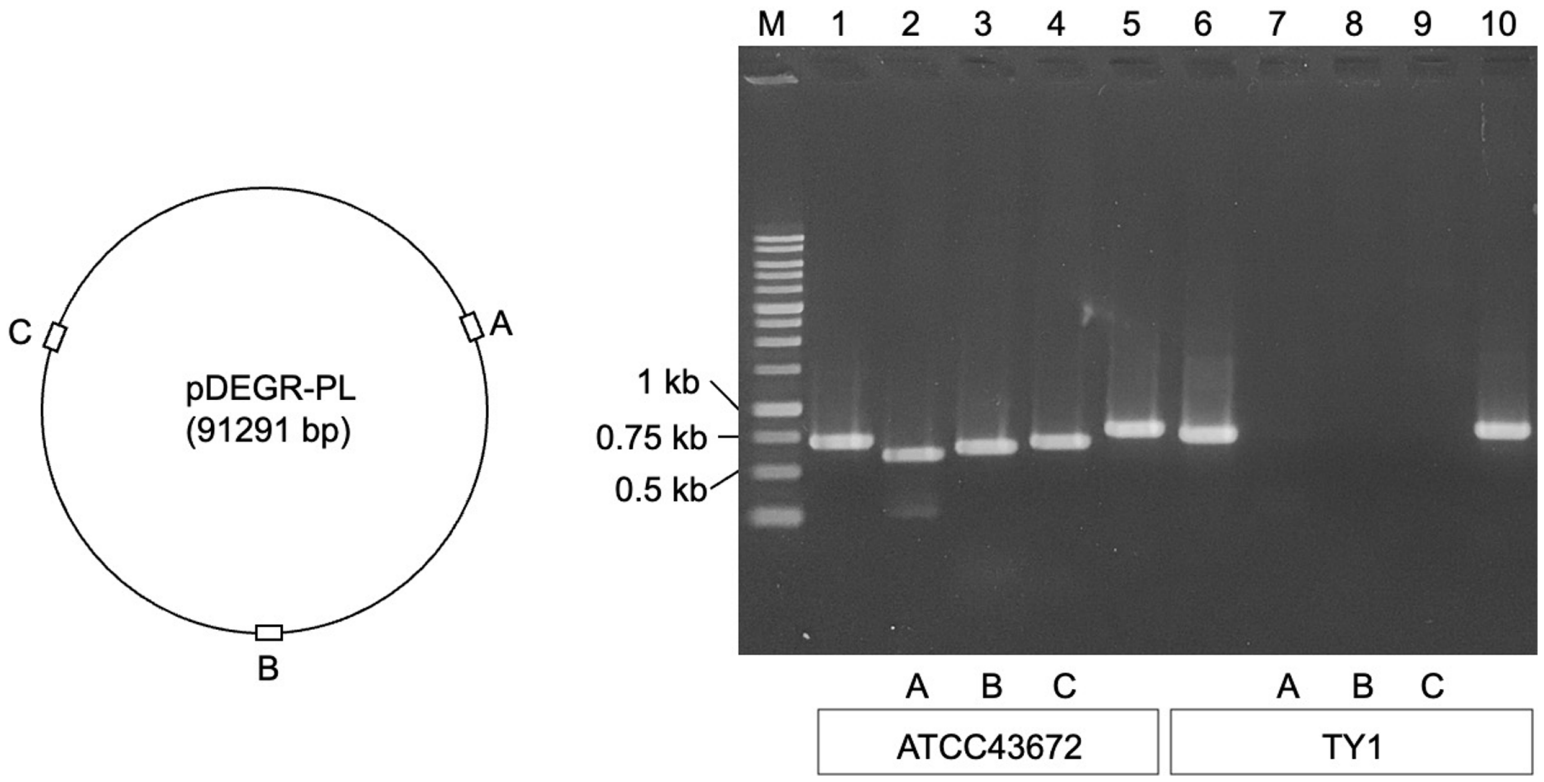
Confirmation of cryptic plasmids pDEGR-PL and pDEGR-3 in *D. grandis.* (Left) Relative locations of DEIGR_400031 **(A)**, DEIGR_400086 **(B)**, and DEIGR_400127 **(C)** in pDEGR-PL. (Right) PCR product detection using agarose gel electrophoresis. M, 1 kb ladder marker (Nippon Genetics Co., Ltd.); lanes 1 and 6, PCR products amplifying the *glnN* gene located on *D. grandis* chromosome; lanes 2 and 7, PCR products amplifying DEIGR_400031 **(A)**; lanes 3 and 8, PCR products amplifying DEIGR_400086 **(B)**; lanes 4 and 9, PCR products amplifying DEIGR_400127 **(C)**; lanes 5 and 10, PCR products amplifying DEIGR_500007 located on pDEGR-3. In lanes 1 to 5, genomic DNA extracted from ATCC43672 (wild type) was used as the PCR template; in lanes 6 to 10, genomic DNA extracted from *D. grandis* strain TY1 was used as the PCR template. The oligonucleotide primer sets used are listed in Supplementary Table 1.

Figure 1 shows the results of agarose gel electrophoresis for the presence of PCR products derived from the glutamine synthetase gene (*glnN*) located on the chromosome of *D. grandis*, DEIGR_400031, DEIGR_400086, and DEIGR_400127 genes located in pDEGR-PL, and the DEIGR_500007 gene located in pDEGR-3. PCR fragments using primer pairs to amplify the *glnN* gene (Figure 1, lanes 1 and 6) and genes located at pDEGR-3 (Figure 1, lanes 5 and 10) were detected in both bacterial isolates that were grown at 40°C and those at 30°C. In contrast, three PCR fragments using primer pairs that amplified different DNA regions of pDEGR-PL were detected in the control genome extracted from the bacterial isolate cultured at 30°C (Figure 1, lanes 2–4) but not in that cultured at 40°C (Figure 1, lanes 7–9). Upon culturing at 40°C, the bacterial isolate lost the plasmid pDEGR-PL but retained pDEGR-3, leading to its designation as *D. grandis* strain TY1.

### 3.2 Generation of *Deinococcus grandis* strain TY3

In addition to high-temperature incubation, rifampicin can eliminate plasmids from microorganisms (Johnston and Richmond, 1970; McHugh and Swartz, 1977). In this experiment, the addition of these chemical agents to the culture medium was combined with incubation at high temperature. To facilitate selecting pDEGR-3 deletion strains from the *D. grandis* TY1 strain, the DEIGR_500008 gene present in pDEGR-3 was replaced with a luciferase gene from deep sea shrimp and a streptomycin resistance gene to generate the *D. grandis* strain TY1Nluc (Section 2.4). Then, luciferase-nonproductive and streptomycin-sensitive colonies were selected as described in Section 2.4. The results of agarose gel electrophoresis are shown in Supplementary Figure 9. This result indicates that the bacterial isolate lacked pDEGR-3Δ8LA of 8,961 bp that was present in strain TY1Nluc and designated as *D. grandis* strain TY3.

### 3.3 Genetic organization of pDEGR-3

**Figure 2:**
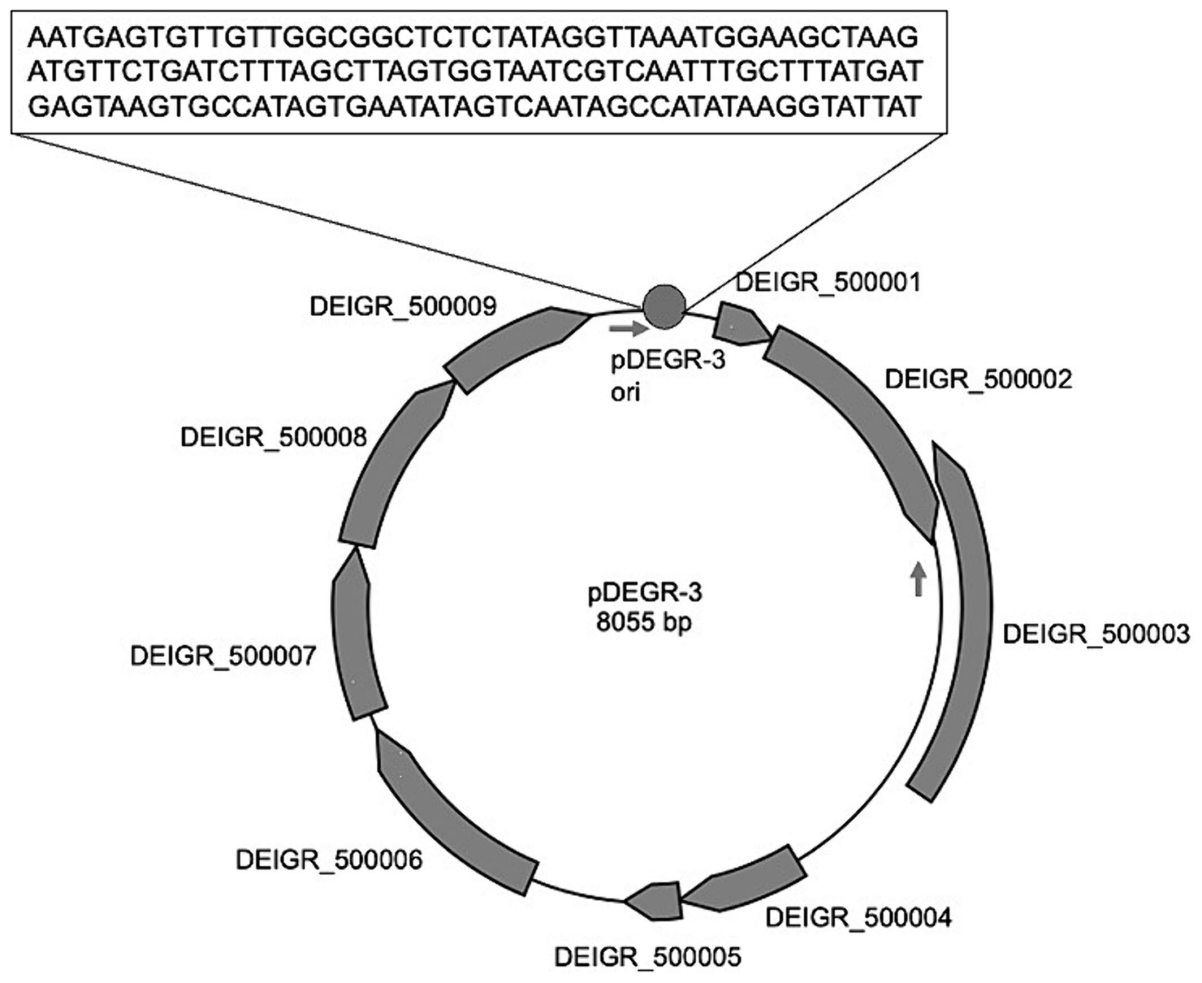
Genetic organization of pDEGR-3. Nine potential open reading frames in pDEGR-3 are depicted as gray boxes; PCR primer (DEGR3F1Kpn and DEGR3R1Kpn) positions for pDEGR-3 replicon amplification are displayed by arrows. Putative pDEGR-3 replication origin is represented as a gray circle. The 139-bp DNA sequence of the putative pDEGR-3 replication origin that shows low %G + C (35%) is presented in a box.

**Figure 3:**
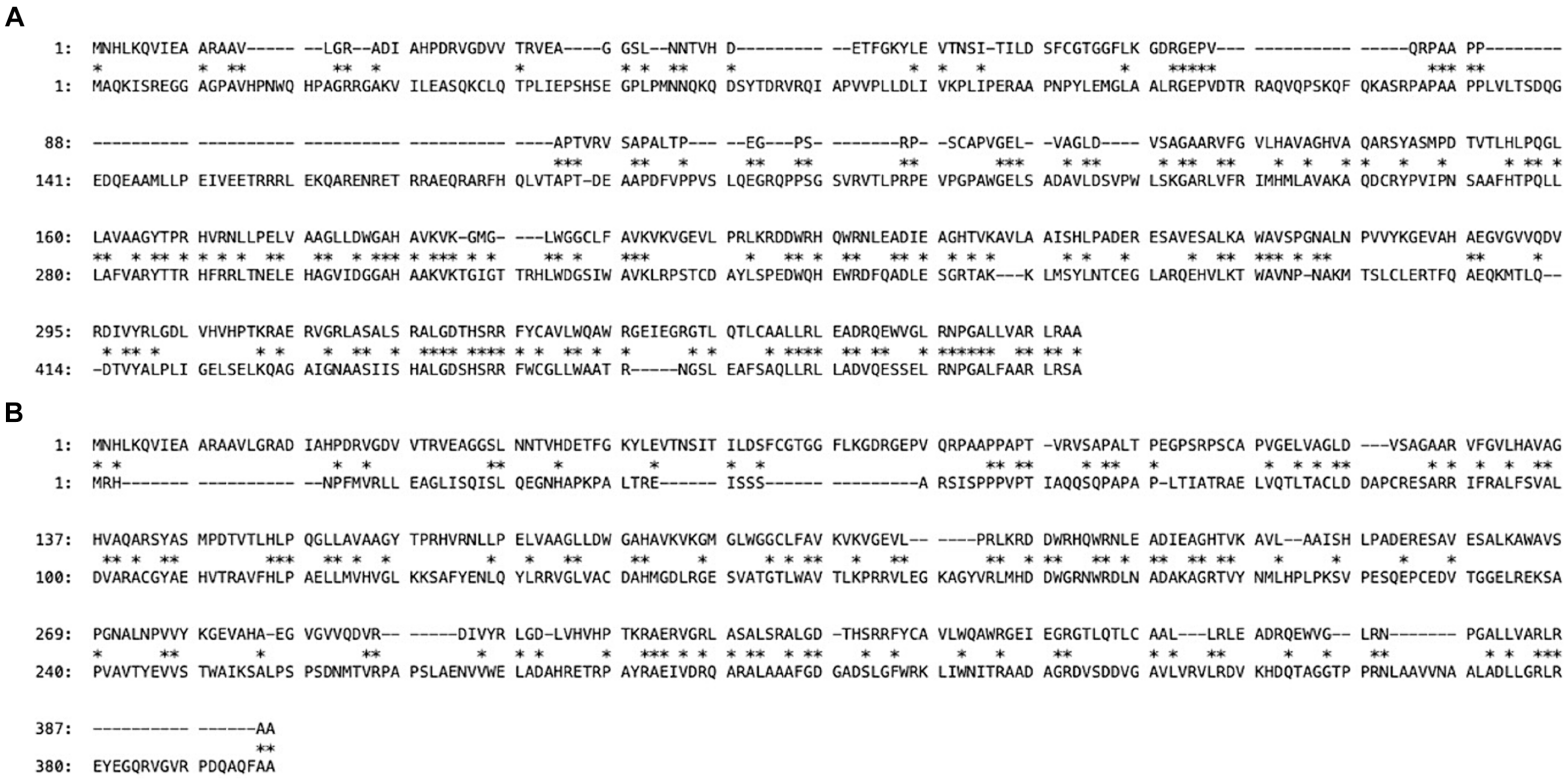
Maximum matching of amino acid sequences of putative replication initiation proteins encoded by two different cryptic plasmids. The upper sequence is from *D. grandis* cryptic plasmid pDEGR-3 and the lower sequences are from different cryptic plasmids of *D. radiopugnans* **(A)** and *D. radiodurans* Sark **(B)**. Sequence alignment gaps are indicated by dashes and the numbers represent coordinates of each protein. Asterisks indicate conserved amino acid residues between the two proteins. Maximum matching was determined using the GENETYX-MAC software version 22.0.1 (GENETYX Co., Ltd., Tokyo, Japan).

Figure 2 shows the genetic organization of the *D. grandis* cryptic plasmid, pDEGR-3. The plasmid contained nine putative open reading frames, of which the protein encoded by DEIGR_500002 (Accession No. GAQ24009) was considered to be the pDEGR −3 replication initiation protein for the following reasons: Figure 3 shows a comparison of this protein with the replication initiation protein of the cryptic plasmid pUE30 from *D. radiopugnans* (Accession No. BAH03365), and that of cryptic plasmid pUE10 of *D. radiodurans* strain Sark (Accession No. AAF44040). The amino acid identity between the replication initiation proteins of pDEGR-3 and pUE30 was 31.7%, and that between the replication initiation proteins of pDEGR-3 and pUE10 was 26.3%. The GC% of a 139-bp non-coding region between DEIGR_500009 (methyltransferase) and DEIGR_500001 (hypothetical protein) was 35.5% and more AT-rich than other plasmid regions because the GC% of the entire pDEGR-3 was 64.9%. The AT-rich region is considered a putative replication origin (Figure 2). Therefore, in this study, the DNA region containing the AT-rich region and DEIGR_500002 was PCR-amplified as a replicon of pDEGR-3 and used to construct a shuttle vector.

### 3.4 Shuttle vector construction

**Figure 4:**
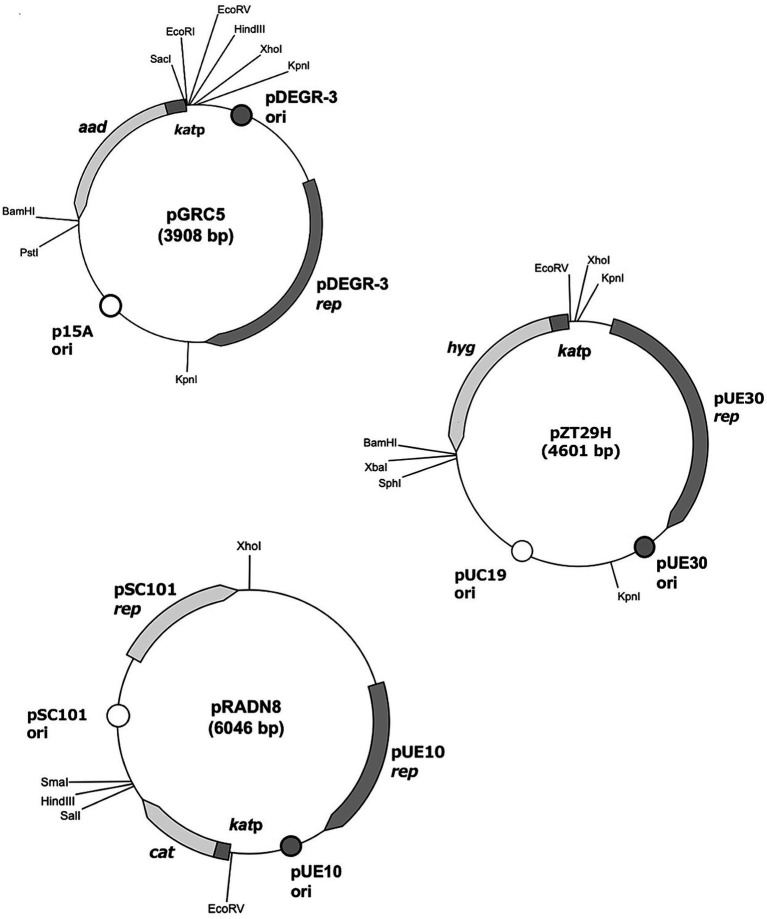
Structure of *E. coli-D. grandis* shuttle vectors. Plasmid pGRC5 consists of the p15A replicon (p15A *ori*) from *E. coli* vector pACYC184, the replication initiation protein-coding gene from *D. grandis* cryptic plasmid pDEGR-3 (pDEGR-3 *rep*), the putative pDEGR-3 replication origin (pDEGR-3 *ori*), and a streptomycin resistance gene (aad). The streptomycin resistance gene is controlled by the modified *D. radiodurans* catalase promoter (*kat*p). pRADN8 contains the pSC101 replicon (pSC101 *ori* and *rep*) from *E. coli* vector pGBM5, the initiation protein-coding gene from *D. radiodurans* Sark cryptic plasmid pUE10 (pUE10 *rep*), the putative pUE10 replication origin (pUE10 *ori*) and a chloramphenicol resistance gene (*cat*). The chloramphenicol resistance gene is controlled by the modified *D. radiodurans* catalase promoter (*kat*p). pZT29 contains the replicon of *E. coli* vector pUC19 (pUC19 *ori*), the initiation protein-coding gene from *D. radiopugnans* cryptic plasmid pUE30 (pUE30 *rep*), the putative pUE30 replication origin (pUE30 *ori*), and a hygromycin resistance gene (*hyg*). The hygromycin resistance gene is controlled by the modified *D. radiodurans* catalase promoter (*kat*p).

The *E. coli*-*D. grandis* shuttle vector pGRC5 was constructed as follows: First, plasmid pKatAAD2L was constructed via ligating plasmid pKatAAD2, which expressed a streptomycin resistance gene (*aad*) controlled by the *D. radiodurans* catalase gene promoter (*kat*p), with the replicon of the *E. coli* vector pACYC184. A DNA fragment containing the replicon of pDEGR-3 of *D. grandis* was PCR-amplified and ligated to pKatAAD2L to yield pGRC5. The second *E. coli*-*D. grandis* shuttle vector, pZT29H, was constructed via changing the selection marker chloramphenicol resistance gene (*cat*) in the plasmid pZT29 to a hygromycin resistance gene (*hyg*). The third *E. coli*-*D. grandis* shuttle vector pRADN8 was constructed as follows: First, plasmid pKatCAT5, which could express a chloramphenicol resistance gene (*cat*) by the *D. radiodurans* catalase gene promoter (*kat*p), and the replicon (pUE10 *rep*) of the *D. radiodurans* Sark strain plasmid pUE10 to yield pRAND7. The plasmid was ligated to *E. coli* vector pGBM5 replicon to yield pRADN8. Structures of the three shuttle vectors are shown in Figure 4, and their DNA sequence information is shown in Supplementary Figures 10–12. Plasmid profiles in *D. grandis* transformants carrying the three shuttle vectors are shown in Supplementary Figure 13. pGRC5 was mainly detected as a monomer in *D. grandis.* The dimer forms of pZT29H and pRADN8 were also detected (Supplementary Figure 13A). Each of the plasmids tested had a single *Xho*I digestion site (Figure 4); the size of the linear plasmids after *Xho*I digestion was in good agreement with the estimated size of monomers (Supplementary Figure 13B).

### 3.5 Plasmid copy number estimation

Relative plasmid copy number was estimated using qPCR assay as described in Section 2.7. The results showed that the relative plasmid copy numbers of pGRC5, pZT29H, and pRADN8 in *D. grandis* transformants were 11, 26, and 5 per genome, respectively (Supplementary Table 2).

### 3.6 Stability of shuttle vectors

In *D. grandis*, DNA fragments (*groE*p*-Nluc* cassette) with the deep sea shrimp luciferase gene placed downstream of *D. radiodurans groES* minimum promoter were inserted into pGRC5, pZT29H, and pRADN8 to express foreign genes and construct expression plasmids pGRC5GN, pZT29HGN, and pRADN8GN, as described in Section 2.8. The expression plasmid profiles in *D. grandis* are shown in Supplementary Figure 14. In lane 1, a band was observed at 4,545 bp derived from pGRC5GN, indicating that the plasmid with pDEGR-3 replicon was present only as a monomer in *D. grandis* strain TY3, similar to the results for pGRC5 shown in Supplementary Figure 13A. In lane 2, bands were observed at 5,250 bp derived from pZT29HGN monomer and 10,500 bp derived from pZT29H dimer. Lane 3 shows a band at 6,683 bp derived from the pRADN8GN monomer and a larger band indicating the plasmid’s multimers. The size of the linear plasmids after *Xho*I digestion was in good agreement with the estimated size of monomers (Supplementary Figure6, 14B).

**Figure 5:**
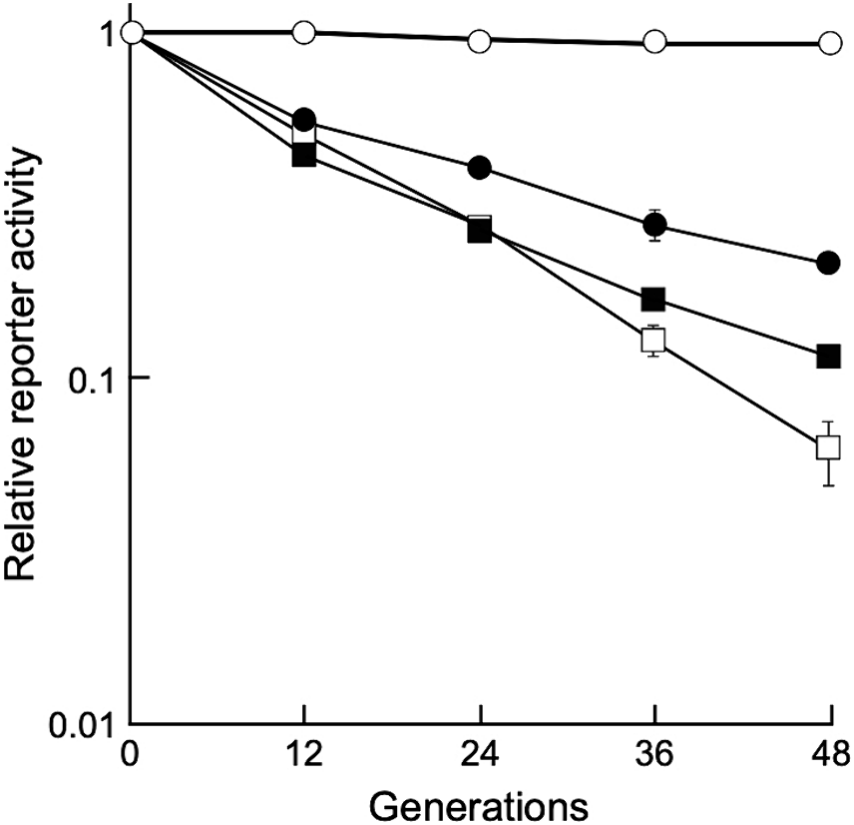
Stability of shuttle vectors under non-selective conditions. Values represent the relative reporter activity at the 0 generation as 1 (mean ± standard error, *n* = 9). Symbols: open circles, *D. grandis* strain TY3 carrying pGRC5GN; open squares, *D. grandis* strain TY3 carrying pZT29HGN; closed circles, *D. grandis* strain TY3 carrying pRADN8GN; closed squares, *D. radiodurans* strain ATCC13939 carrying pRADN8GN.

To determine whether the shuttle vectors could stably replicate under non-selective conditions, *D. grandis* transformants carrying pGRC5GN, pZT29HGN, and pRADN8GN were grown in non-selective broth until 48 generations by repeated dilution and incubation, and relative chemiluminescence intensity was determined as described in Section 2.8. As shown in Figure 5, *D. grandis* transformants carrying pGRC5GN exhibited constant luminescence intensity until 48 generations, whereas the luminescence intensity of *D. grandis* transformants carrying pZT29HGN or pRADN8GN decreased as the generation progressed. This result was in good agreement with the analysis of plasmid retention in culture under non-selective conditions using agarose gel electrophoresis (Supplementary Figure 14B).

### 3.7 Induction of gene expression via ultraviolet irradiation

**Figure 6:**
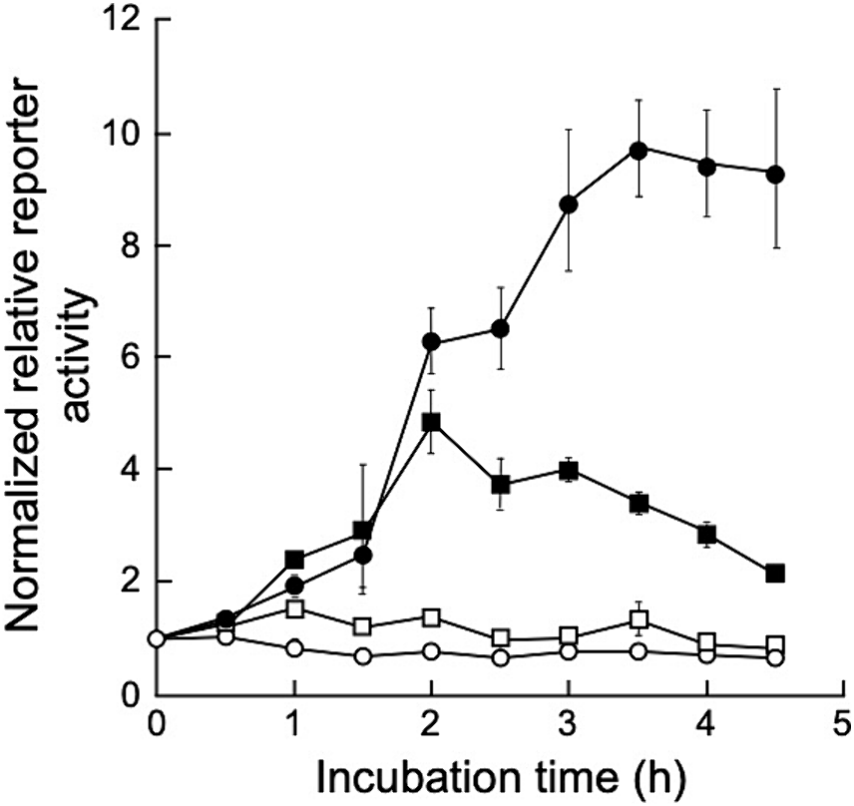
Normalized relative reporter activity in pRDR-NlucR-transformants exposed to different UV-C doses. Values represent the relative reporter activity normalized to activity at the 0-time point for each dose as 1 (mean ± standard error, *n* = 3). Symbols: open circles, 0 J/m^2^; open squares, 20 J/m^2^; closed squares, 50 J/m^2^; closed circles, 150 J/m^2^.

To determine gene expression induction via UV irradiation in *D. grandis*, a dual-luciferase reporter plasmid, pRDR-NlucD, was constructed as described in Section 2.9. This plasmid is based on shuttle vector pGRC5. It contains the deep sea shrimp luciferase gene (*Nluc*) with *D. grandis ddrO* promoter (*ddrO*p) and firefly luciferase gene (*luc*+) with the *D. radiodurans groES* minimal promoter (*groE*p). This plasmid was introduced into TY3, which already contained pZT29H, and luciferase activity was monitored after UV irradiation. Figure 6 shows the normalized relative reporter activity in the pRDR-NlucR-transformants exposed to different UV-C doses. When irradiated with 20 J/m^2^ of UV-C, the maximum value (approximately 1.5 times the value immediately after irradiation) was observed 1 h post-irradiation incubation; at 50 J/m^2^, (approximately 4.9 times the value immediately after irradiation) 2 h post-irradiation incubation; at 150 J/m^2^, (approximately 9.8 times the value immediately after irradiation) 3.5 h post-irradiation incubation (Figure 6). Thus, using pRDR-NlucD enabled gene expression induction in TY3 via UV irradiation and revealed the irradiation dose dependence of gene expression levels.

### 3.8 Transformation of *Deinococcus radiodurans* using pGRC5, pZT29H, and pRADN8

We attempted to introduce the newly developed shuttle vector into *D. radiodurans*. As expected, *D. radiodurans* transformants were generated with pRADN8. Relative plasmid copy numbers of pRADN8 in *D. radiodurans* transformants were 15 per genome (Supplementary Table 2). *D. radiodurans* transformants carrying pRADN8GN exhibited approximately 15.5% of the initial luminescence intensity at the 48 generation (Figure 5). Similar to previous studies, we were unable to generate *D. radiodurans* transformants using pUE30-based shuttle vector pZT29H (Satoh et al., 2009). Likewise, *D. radiodurans* transformants could not be generated using pGRC5.

## 4 Discussion

### 4.1 *Deinococcus grandis* genome organization

We previously analyzed the draft and whole genome sequences of *D. grandis* (Satoh et al., 2016; Shibai et al., 2019); both are available in public genome databases (GenBank Assembly ID: GCA_001485435.1 and GCA_009177165.1). Table 2 summarizes results of the two analyses. Four circular and three linear contigs were registered in the draft genome analysis; four circular DNAs were registered in the whole genome sequence analysis: chromosome, pDEGR-1, pDEGR-2, and pDEGR-3. Next-generation sequencing analyses were performed using genomic DNA extracted from the *D. grandis* type strain. However, whole genome sequence analysis did not detect one of the circular contigs (BCMS01000006) found in the draft genome analysis. Previous studies have not described a specific name for this plasmid; hence, it was referred to as pDEGR-PL. The laboratory stock of the *D. grandis* type strain used in draft genome sequencing was used in this study.

**Table 2:** Summary of *D. grandis* genome analysis.

GenBank assembly ID	GCA_001485435.1	GCA_009177165.1
Locus tag prefix	DEIGR	DEGR
Annotated Genes	4,104	4,041
Genome		
Chromosome	BCMS01000001 (Circular) 3,250,361 bp	AP021849 (Circular) 3,241,502 bp
pDEGR-1	BCMS01000002(Circular) 389,340 bp	AP021850 (Circular) 389,567 bp
pDEGR-2	BCMS01000003 (Linear) BCMS01000004(Linear) BCMS01000005(Linear) 353,450 bp	AP021851 (Circular) 373,915 bp
pDEGR-PL	BCMS01000006 (Circular) 91,291 bp	
pDEGR-3	BCMS01000007 (Circular) 8,055 bp	AP021852 (Circular) 8,055 bp
References	Satoh et al. (2016)	Shibai et al. (2019)

In this study, we generated two strains: TY1 that lacked pDEGR-PL, and TY3 that lacked both pDEGR-PL and pDEGR-3. Plasmid pDEGR-PL was deleted via cultivation at 40°C, the upper limit of the permissible temperature, whereas pDEGR-3 was stable against heat treatment. Plasmid pDEGR-PL was undetected via whole genome sequencing in a previous study (Shibai et al., 2019). As the largest coding DNA sequence of the undetected circular contig was similar to that of the phage tail protein, this circular DNA was assumed to be a mobile genetic factor and thus lost prior to whole-genome sequencing. In this study, pDEGR-PL was believed to be easily cured from the *D. grandis* type strain owing to the instability of the plasmid against heat. Thus, pDEGR-PL may have been transferred from other psychrophiles to *D. grandis*.

### 4.2 Shuttle vectors

When multiple plasmid vectors are used, homologous recombination reactions between them must also be suppressed. To overcome this, we constructed a shuttle plasmid as a replication initiation region of three plasmids from *E. coli* with different nucleotide sequences and three plasmids from *Deinococcus* spp. ligated together, thereby avoiding plasmid instability owing to nucleotide sequence homology. The replication initiation regions of the three plasmids from *E. coli* used in this study are known to coexist without interfering with each other (Bartolome et al., 1991; Manen et al., 1997). However, the compatibility of replication initiation regions of the three plasmids from *Deinococcus* spp. is unclear. In this study, we experimentally demonstrated that, despite the limited homology of these replication initiation proteins (Figure 3), the plasmids did not interfere with each other and could stably coexist in *D. grandis* (Supplementary Figure 13).

Each shuttle vector carries a different antibiotic resistance gene as a selection marker, which ensures the plasmid presence in the bacteria in a selective culture medium, extraction and purification of plasmids from the bacteria, and introduction of plasmids into the bacteria via transformation. The antibiotic resistance genes in the three shuttle vectors are regulated by the *D. radiodurans* catalase gene promoter (*kat*p), and this promoter region (123 bp) is the same sequence in the three shuttle vectors. Because homologous recombination between different shuttle vectors did not occur under the experimental conditions, homologous recombination in *D. grandis* is thought to require a longer homologous DNA size. In a previous study, *D. grandis* could not be transformed with the pUE10-based shuttle vector pRADN1 (Satoh et al., 2009); however, in the present study, *D. grandis* was transformed with pRADN8. This difference is because of the different plasmid DNA sequences, and likely depends on the presence or absence of plasmid DNA cleavage by the restriction enzymes present in *D. grandis*.

pGRC5, pZT29H, and pRADN8 have pUC19-derived multiple cloning sites upstream and downstream of the antibiotic marker gene, and some of these restriction sites can be used as cloning sites. However, in the process of constructing the shuttle vector, additional recognition sites were created at other sites in the plasmid, making some of the restriction sites in the multiple cloning sites unusable as cloning sites. By appropriately modifying the nucleotide sequence of the newly developed shuttle vectors, the unusable cloning sites can be restored in the future.

### 4.3 Plasmid stability

Among the three plasmids, the copy number per genome for pRADN8 was 5. The propensity of pRADN8 and its derived plasmids to form multimeric forms in *D. grandis* may be related to its low copy numbers (Field and Summers, 2011; Crozat et al., 2014). The pUE10-based shuttle vector pRAD1 was shown to be unretained and shed by *D. radiodurans* under culture conditions with no selection pressure (Meima and Lidstrom, 2000). Plasmid pRADN8 is presumed to be unstably retained by *D. grandis* under culture conditions without selection pressure. In this study, we determined plasmid stability in *D. grandis* transformants carrying plasmids with the *groEp-Nluc* cassette (Figure 5). As expected, pGRC5 showed high stability in *D. grandis* strain TY3, whereas pRADN8 showed low stability.

pUE30-based pZT29H was retained in *D. grandis*, mainly as a monomer and partly as a dimer. The mode of presence of this plasmid was similar to that of pZT29 in a previous study (Satoh et al., 2009). The pUE30-based shuttle vector pZTGL93 that contains the *D. radiodurans groES* minimal promoter and firefly luciferase gene has been shown to be stably retained by *D. grandis* wild-type strain for 48 generations under culture conditions without selection pressure (Satoh et al., 2009). Unlike this, *D. grandis* strain TY3 transformants carrying pZT29HGN exhibited approximately 6% of the initial luminescence intensity at the 48 generation (Figure 5). We suggest that plasmids present in wild strain but absent in strain TY3, namely pDEGR-PL or pDEGR-3, may encode genes responsible for the stability of pUE30-based shuttle vectors.

### 4.4 Expression plasmid

In this study, we constructed pRDR-NlucD, in which the luciferase gene was controlled by the *ddrO* promoter in *D. grandis*. We showed that UV irradiation increased luciferase activity in pRDR-NlucD-transformants (Figure 6). The *ddrO* promoter region contains an operator sequence called the radiation desiccation-responsive motif (RDRM) that binds to the repressor DdrO protein and represses its gene expression. The DdrO protein is cleaved by the protease activity of the PprI protein activated by DNA damage stress, de-repressing the downstream gene expression (Ishino and Narumi, 2015). This DNA damage stress response mechanism by PprI and DdrO is highly conserved in several *Deinococcus* spp., including *D. grandis*, for which genome analysis has been completed (Lim et al., 2019). The dual-luciferase reporter plasmid pRDR-NlucD coexisted with another type of shuttle vector, pZT29H. In the future, cloning genes that affect the DdrO/PprI stress response mechanism into pZT29H could provide a tool to elucidate the molecular mechanisms of the DdrO/PprI-dependent stress response in *D. grandis*.

In addition to the *ddrO* promoter, promoters that are upstream of the *pprA*, *ddrA*, *ddrB*, and *gyrB* genes and have RDRM operator sequences in their vicinity can be used as promoters to induce the expression of foreign genes in *D. grandis* following UV irradiation (Narasimha and Basu, 2021). Their activity in foreign gene expression in *D. grandis* should be investigated in the future. Promoters of *DR1261*, *rpmB*, and *dnaK* have been reported to allow constitutively high expression of these genes in *D. radiodurans* (Chen et al., 2019). Therefore, these promoters should be tested in the future to determine the similarity of their effects on *D. grandis*. It will also be desirable to develop vectors for the expression of recombinant genes in the periplasm of enlarged *D. grandis* spheroplasts in the future.

### 4.5 Concluding remarks

Thus this study reports on the successful development of a novel system that stably introduced and retained expression plasmids for *D. grandis*. From the wild-type strain, two cryptic plasmids were eliminated to generate the strain TY3. Three shuttle vector plasmids, pGRC5, pRADN8, and pZT29H, were shown to coexist in *D. grandis.* These plasmids are expected to facilitate the removal of toxic materials and produce useful compounds in *D. grandis*.

## Data availability statement

The nucleotide sequences of pGRC5, pZT29H, and pRADN8 has been assigned in the DDBJ/EMBL-Bank/GenBank Accession Nos. LC801619, LC801620, and LC801621, respectively.

## Author contributions

MS: Data curation, Investigation, Methodology, Writing – original draft, Writing – review & editing. TS: Investigation, Methodology, Writing – original draft, Writing – review & editing. KK: Investigation, Methodology, Writing – original draft, Writing – review & editing. MY: Conceptualization, Supervision, Writing – original draft, Writing – review & editing. IN: Conceptualization, Funding acquisition, Supervision, Writing – original draft, Writing – review & editing.
