# Supplementary material for: Developing a new host-vector system for Deinococcus grandis
Source: Front Microbiol. 2024 May 28;15:1387296. doi: 10.3389/fmicb.2024.1387296 (PMC11165121; doi:10.3389/fmicb.2024.1387296)
Supplement: Supplementary file 3 [file Data_Sheet_1.DOCX]

Supplementary Material


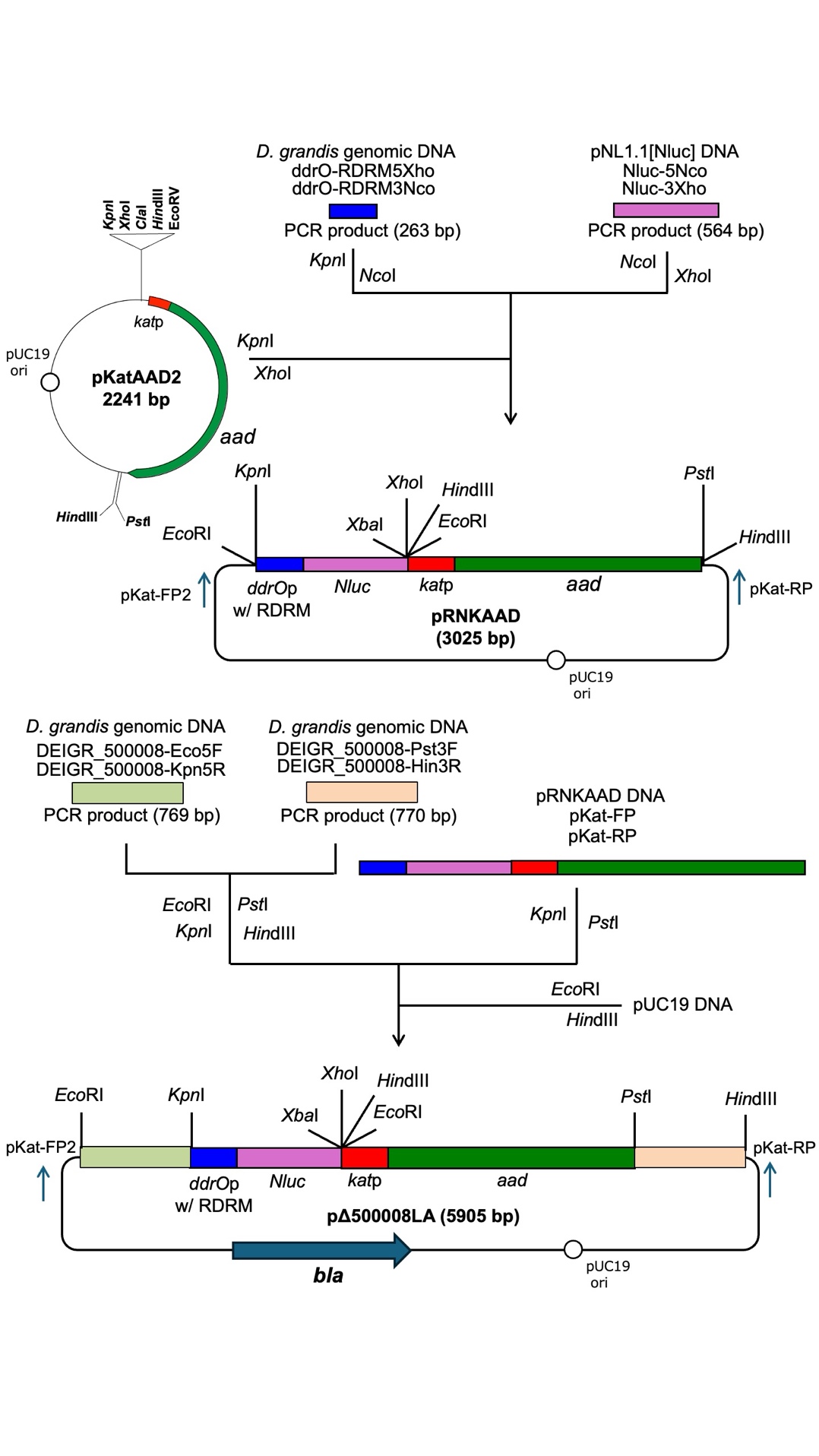


**Supplementary Figure 1.** Construction of plasmid pΔ500008LA.


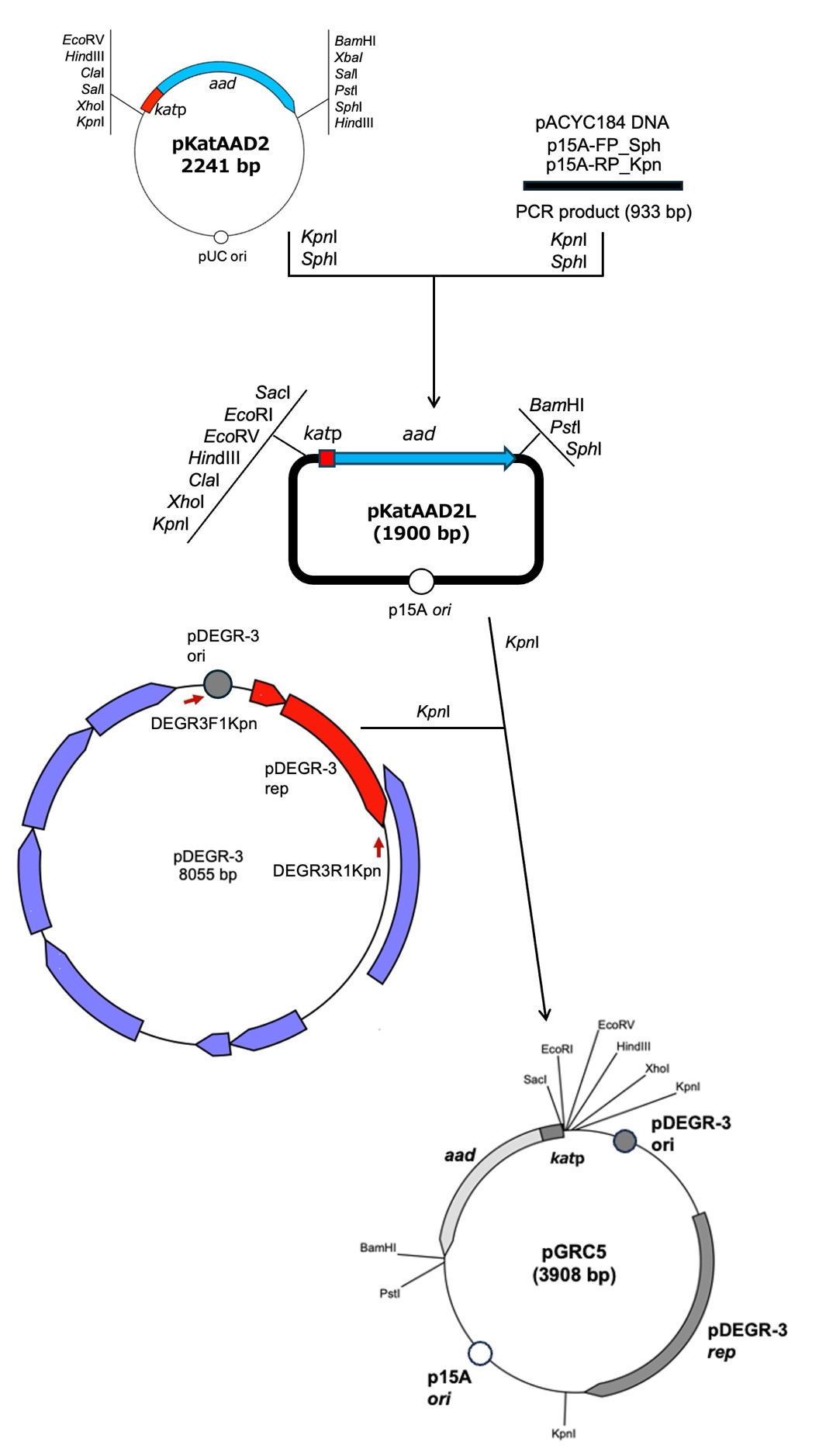


**Supplementary Figure 2.** Construction of plasmid pGRC5.


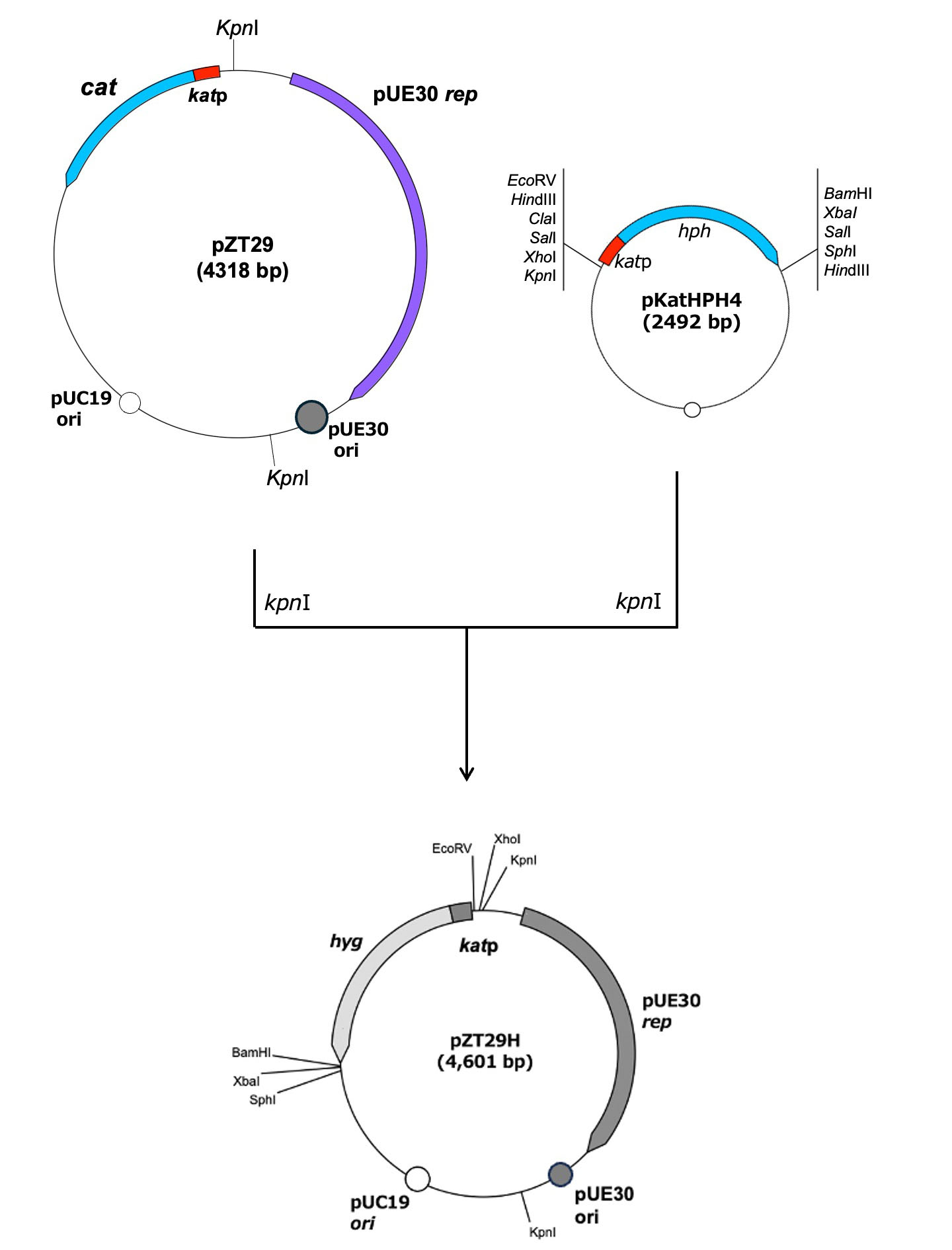


**Supplementary Figure 3.** Construction of plasmid pZT29H.


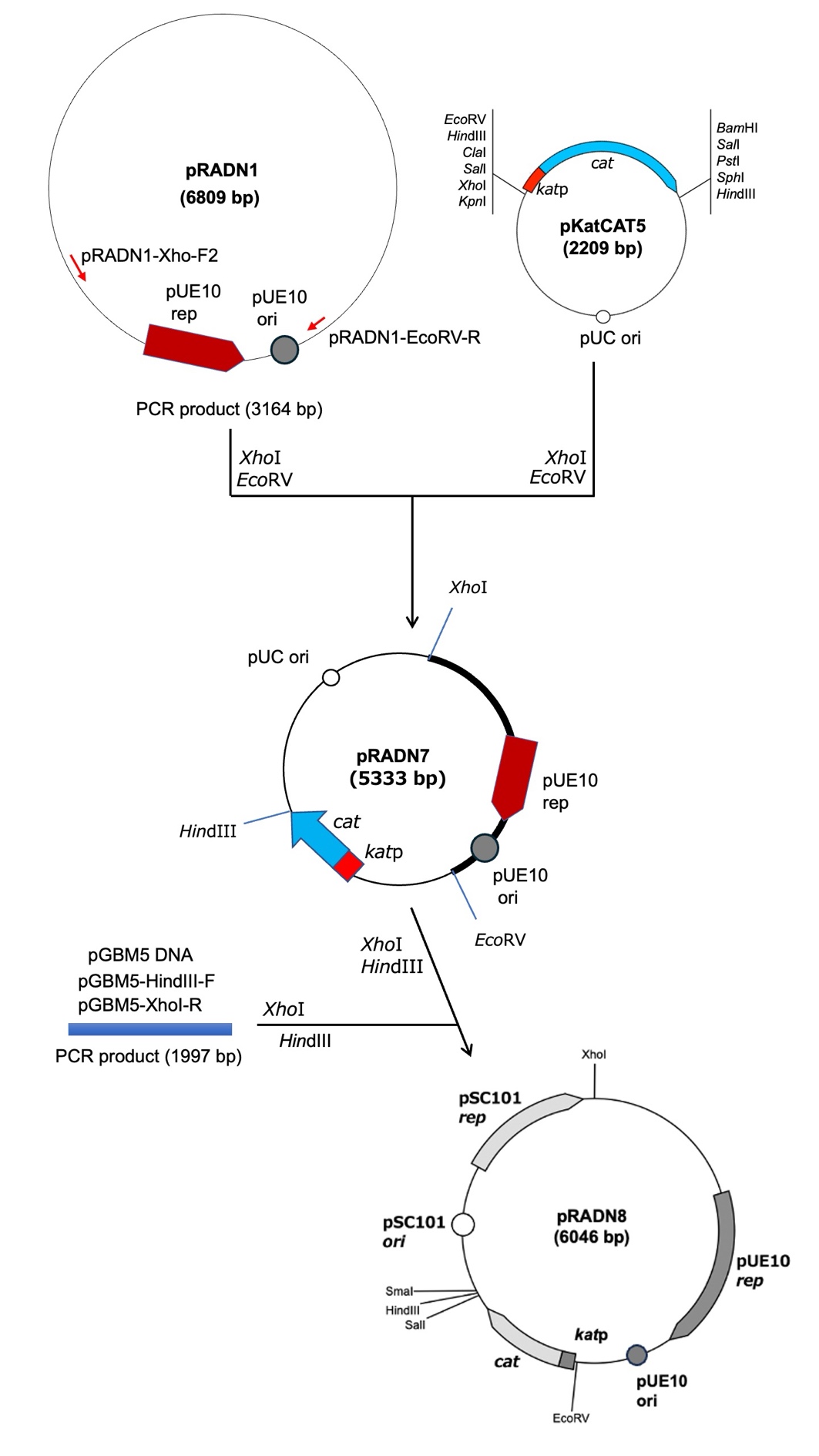


**Supplementary Figure 4.** Construction of plasmid pRADN8.


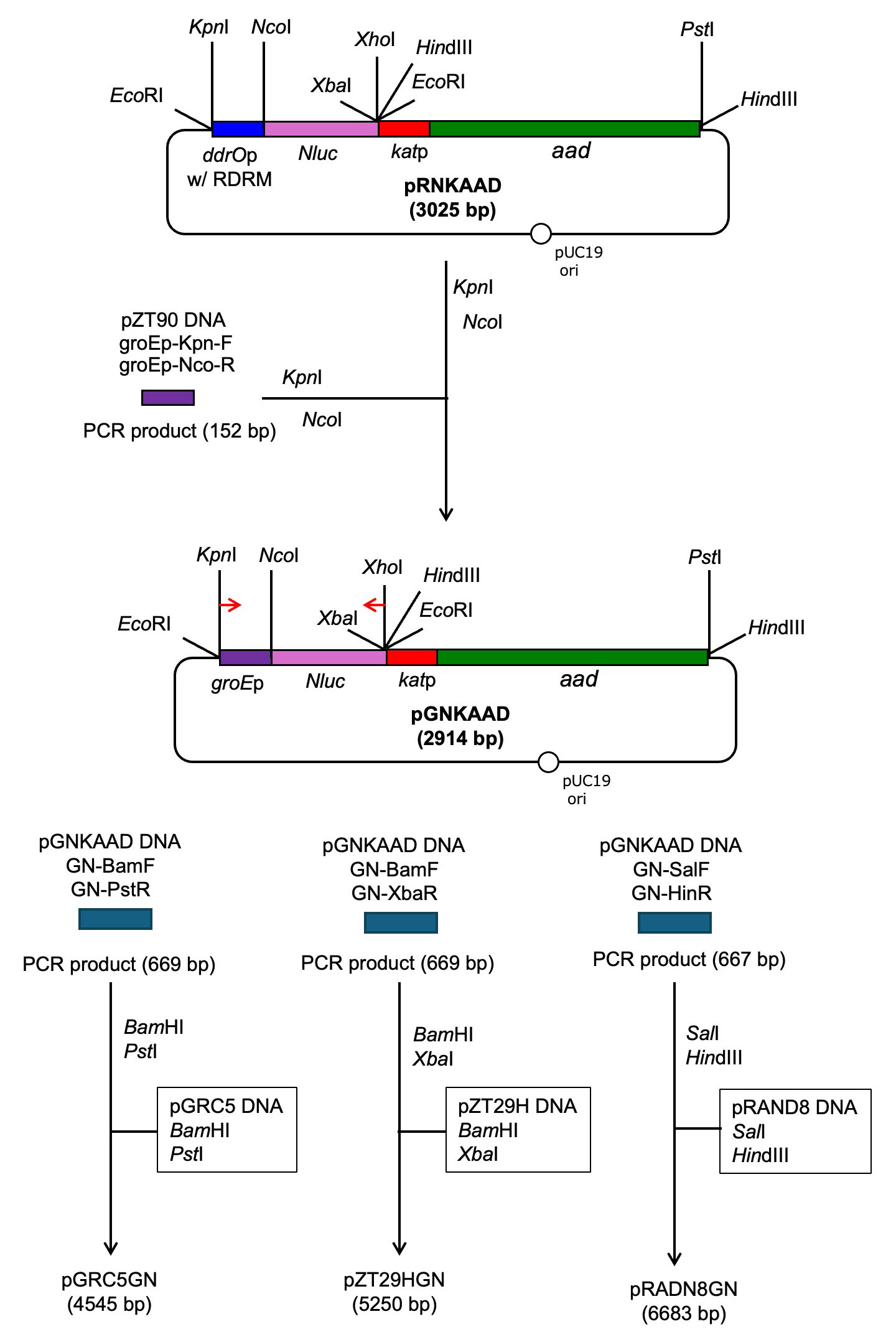


**Supplementary Figure 5.** Construction of plasmid pGNKAAD.

**
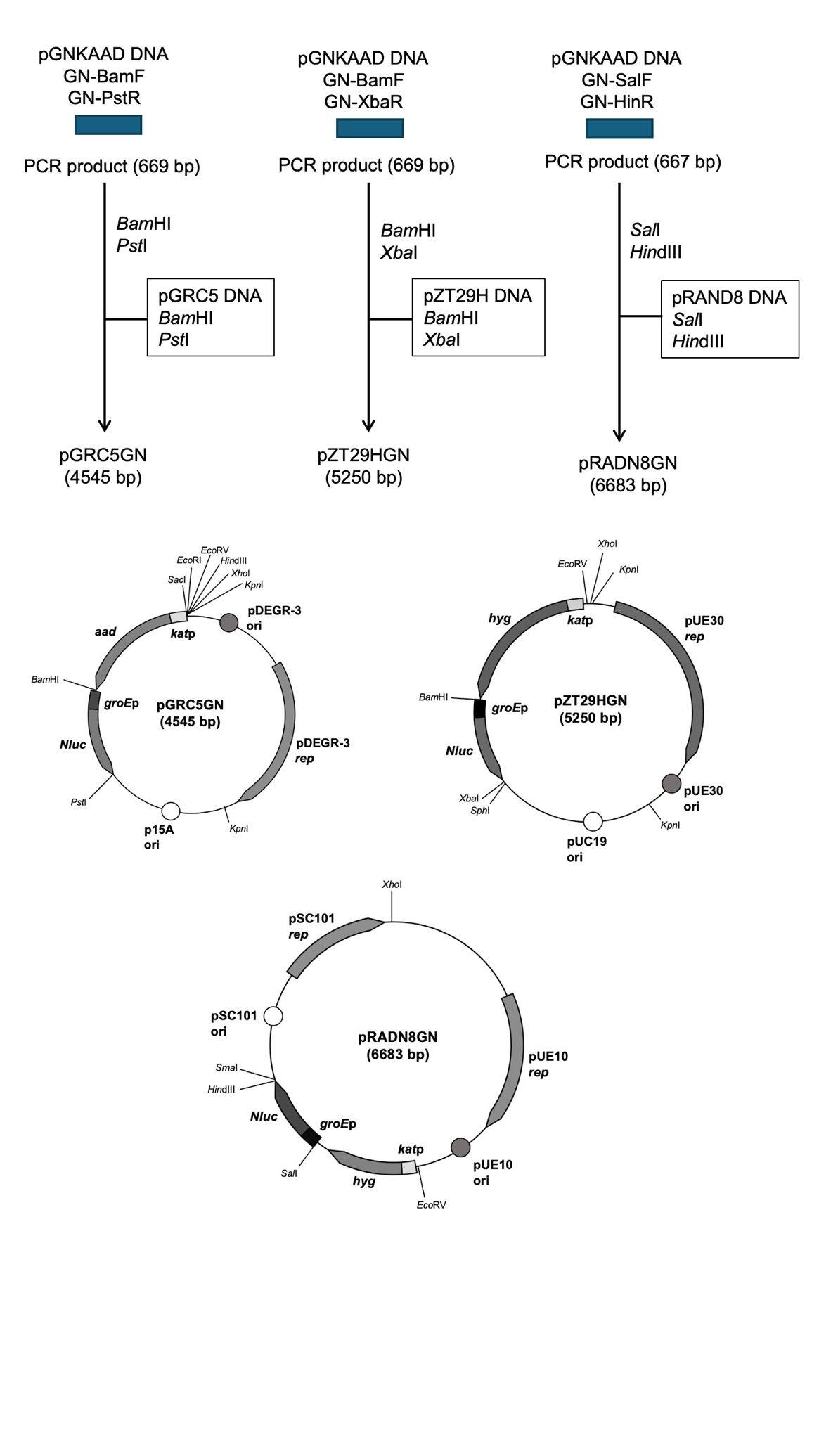
**

**Supplementary Figure 6.** Construction of plasmids pGRC5GN, pZT29HGN, and pRADN8GN.


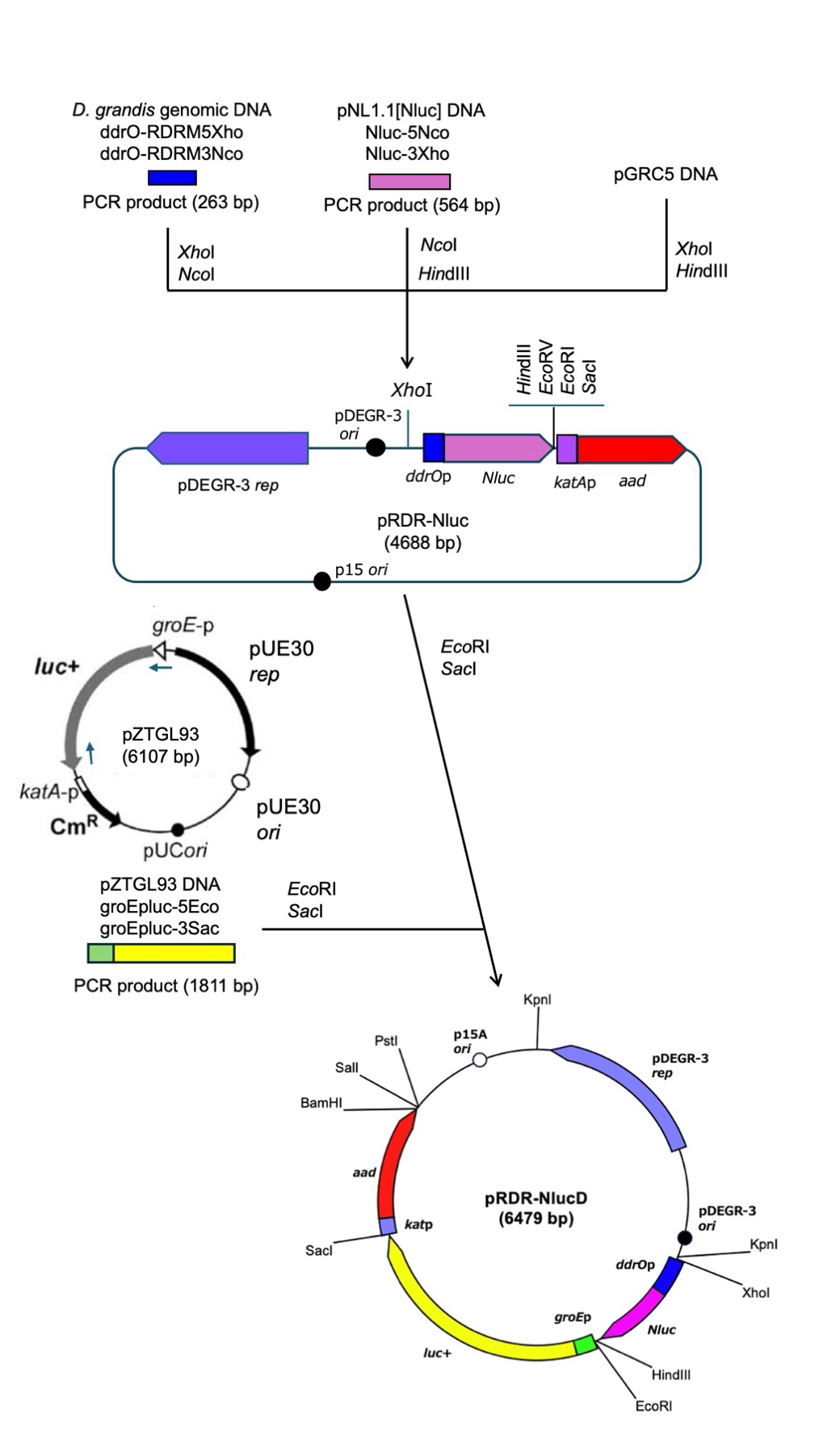


**Supplementary Figure 7.** Construction of plasmid pRDR-NlucD.

Plasmid pRDR-NlucR contains two replicons, p15A and pDEGR-3, and three genes, including deep sea shrimp luciferase (*Nluc*) under the control of *D. grandis ddrO* promoter and firefly luciferase (*luc*+) under the control of *D. radiodurans groES* minimal promoter (*groE*p), as well as a streptomycin resistance gene (*aad*) controlled by *D. radiodurans* catalase promoter (*kat*p).


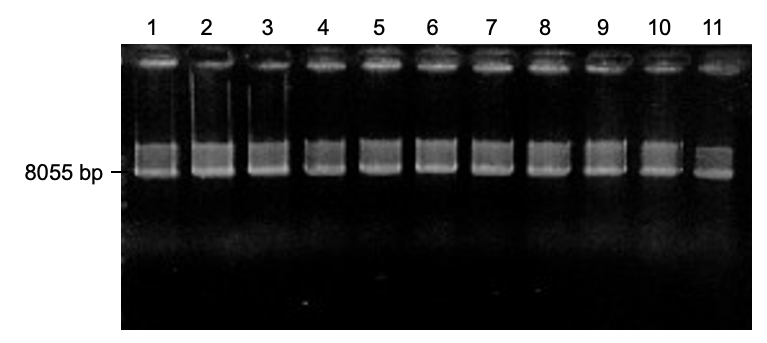


**Supplementary Figure 8.** Confirmation of cryptic plasmid pDEGR-3 in *D. grandis*.

Plasmid extracts from colonies obtained by incubation at 40°C (lanes 1 to 10) and from controls not incubated at 40°C (lane 11) were subjected to agarose gel electrophoresis.


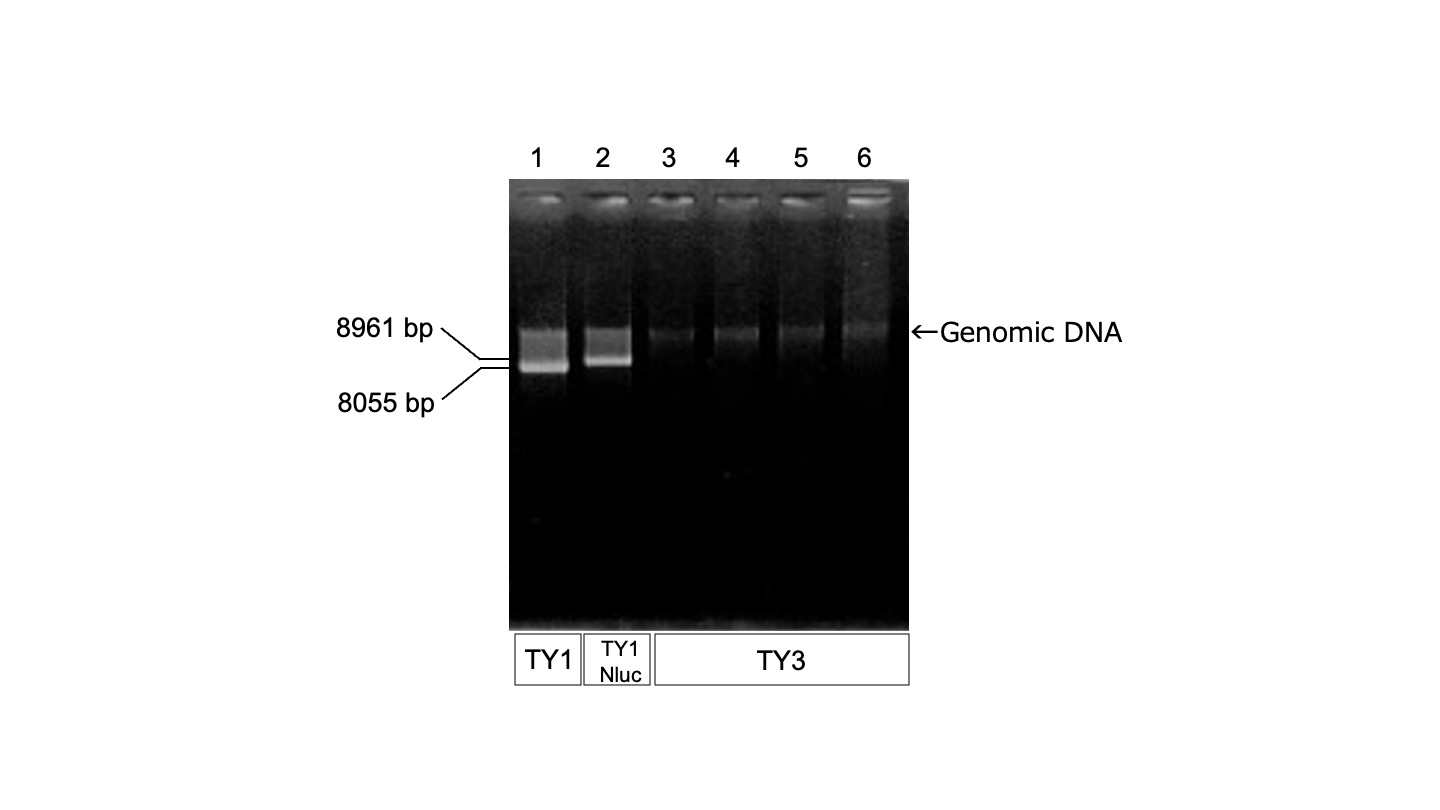


**Supplementary Figure 9.** Confirmation of cryptic plasmid pDEGR-3 in *D. grandis*

Plasmid DNA from TY1 (lane 1), TY1Nluc (lane 2), and TY3 (lanes 3–6) were subjected to agarose gel electrophoresis. Contamination with genomic DNA caused a faint signal in lanes 3–6. Plasmid pDEGR-3 of 8055 bp (lane 1) was found from the strain TY1 grown at 30°C, and plasmid pDEGR-3Δ8LA of 8961 bp (lane 2) from the strain TY1Nluc grown at 30°C, whereas no plasmid was detected from the luciferase nonproductive and streptomycin-sensitive isolate from strain TY1Nluc grown at 40°C (lanes 3 to 6).

ACTTTGACGA TCTTGGGGGA ATGAGTGTTG TTGGCGGCTC TCTATAGGTT AAATGGAAGC 60

TAAGATGTTC TGATCTTTAG CTTAGTGGTA ATCGTCAATT TGCTTTATGA TGAGTAAGTG 120

CCATAGTGAA TATAGTCAAT AGCCATATAA GGTATTATCT GGTGCGATTT CGCGCACTTC 180

CCGGGTACTG GTAACCTCCT ACAAGACCTG CAATTTATGC AGGTGCATGC CCGACATACA 240

CATCCTGTGG GGTGACACTA CAGTGTCATT TTTGCCGTCC TGCACGACGT TGTAGGATTA 300

AATTAGCTCA CAACGTACAA GTGCTTGTTG ATTGCGGCGA GTGGAGATAC AGTGGTGGCA 360

GTTGGGCCGC CATGCCTGAC AACACAAAAG CGCGAAGCAC ACCCGTCCTG GCAGACTCAT 420

GGTGTGCTTC TCCCGAAGCG AACGCGGCAC GTTCGCGGCC ATGTCCGTTC TTGGCCTCAG 480

ATTACACGTG TCCCGCTTTG AGGACAAGTG GGACTGACCT TGTTCATTTT GAGGTTGAGA 540

ACGCGCTCTG GGAGCGGATT CTCTTGGATA CGAACGTGTT TCGGCTTTCT CTGGCTCTAG 600

TCGGTCTAGT TGCCTTCATC GCCCTCTTAG TTGCCACGGT GATCCTGGCG AAGGCAGGAC 660

AGCCCATGTG GGCGGCCTTC ACGGGAACTG GTGCGGTTGT CGTTTGCGTT GGTGCGCTGT 720

ACCGCCTGCT GCTAACACTA GGGCAGCCTG CATGAACCAC CTAAAGCAGG TCATCGAGGC 780

GGCCCGCGCG GCTGTTCTCG GGCGTGCTGA CATCGCCCAT CCTGACCGGG TGGGCGATGT 840

TGTCACGCGT GTTGAAGCGG GCGGCAGCCT GAACAACACC GTTCACGACG AAACCTTCGG 900

GAAGTACCTG GAAGTGACGA ACAGCATCAC GATCCTCGAC TCTTTCTGCG GCACCGGCGG 960

CTTCCTGAAG GGAGATCGTG GAGAACCCGT ACAGCGACCC GCTGCGCCGC CTGCGCCGAC 1020

CGTGCGGGTG TCTGCCCCTG CCCTAACCCC CGAGGGGCCT TCACGGCCTT CCTGTGCGCC 1080

CGTGGGGGAG CTGGTCGCGG GCCTCGACGT GAGCGCCGGG GCAGCGCGGG TCTTCGGAGT 1140

TCTACACGCC GTTGCCGGTC ATGTCGCGCA GGCCCGTTCC TACGCCTCGA TGCCGGACAC 1200

CGTGACCCTC CATCTCCCGC AGGGCCTCTT GGCGGTCGCG GCGGGGTACA CGCCCCGCCA 1260

CGTTCGCAAC CTGCTGCCCG AGCTGGTCGC AGCGGGGCTG CTCGACTGGG GAGCGCACGC 1320

CGTGAAGGTC AAGGGCATGG GCCTGTGGGG CGGGTGCCTC TTCGCGGTGA AGGTCAAGGT 1380

GGGTGAGGTA CTACCCCGCC TGAAGCGCGA CGACTGGCGT CACCAGTGGC GCAACCTGGA 1440

GGCGGACATC GAGGCCGGGC ACACCGTCAA GGCGGTGCTG GCGGCCATTT CCCATTTACC 1500

TGCCGATGAG CGAGAGAGCG CGGTGGAAAG CGCCCTAAAA GCGTGGGCCG TTAGCCCTGG 1560

AAATGCCTTG AACCCCGTTG TTTATAAAGG GGAAGTCGCA CACGCGGAGG GTGTCGGTGT 1620

CGTCCAGGAC GTGCGGGACA TCGTGTACCG GCTGGGTGAT CTGGTCCACG TCCACCCGAC 1680

CAAGCGGGCG GAACGAGTTG GACGGCTGGC GTCGGCCTTG AGCCGGGCGC TCGGGGACAC 1740

ACACTCCCGC CGGTTCTACT GCGCGGTGCT GTGGCAGGCG TGGCGCGGTG AGATCGAGGG 1800

GCGCGGGACG TTGCAGACGC TCTGCGCCGC CCTCTTGCGC CTTGAGGCTG ACCGGCAGGA 1860

GTGGGTCGGA CTGCGGAACC CCGGTGCCCT CCTGGTGGCC CGCCTCAGGG CCGCGTAACG 1920

CCCGGCTGAG GCTTGCCCCG CCCCGAACTC CACCGCGCAC CGGCTCGCGC CTCTGCTGGG 1980

GCATGAGCCG GGTGGTCATC ATGGTACCAC AACTTATATC GTATGGGGCT GACTTCAGGT 2040

GCTACATTTG AAGAGATAAA TTGCACTGAA ATCTAGAAAT ATTTTATCTG ATTAATAAGA 2100

TGATCTTCTT GAGATCGTTT TGGTCTGCGC GTAATCTCTT GCTCTGAAAA CGAAAAAACC 2160

GCCTTGCAGG GCGATTTTTC GAAGGTTCTC TGAGCTACCA ACTCTTTGAA CCGAGGTAAC 2220

TGGCTTGGAG GAGCGCAGTC ACCAAAACTT GTCCTTTCAG TTTAGCCTTA ACCGGCGCAT 2280

GACTTCAAGA CTAACTCCTC TAAATCAATT ACCAGTGGCT GCTGCCAGTG GTGCTTTTGC 2340

ATGTCTTTCC GGGTTGGACT CAAGACGATA GTTACCGGAT AAGGCGCAGC GGTCGGACTG 2400

AACGGGGGGT TCGTGCATAC AGTCCAGCTT GGAGCGAACT GCCTACCCGG AACTGAGTGT 2460

CAGGCGTGGA ATGAGACAAA CGCGGCCATA ACAGCGGAAT GACACCGGTA AACCGAAAGG 2520

CAGGAACAGG AGAGCGCACG AGGGAGCCGC CAGGGGGAAA CGCCTGGTAT CTTTATAGTC 2580

CTGTCGGGTT TCGCCACCAC TGATTTGAGC GTCAGATTTC GTGATGCTTG TCAGGGGGGC 2640

GGAGCCTATG GAAAAACGGC TTTGCCGCGG CCCTCTCACT TCCCTGTTAA GTATCTTCCT 2700

GGCATCTTCC AGGAAATCTC CGCCCCGTTC GTAAGCCATT TCCGCTCGCC GCAGTCGAAC 2760

GACCGAGCGT AGCGAGTCAG TGAGCGAGGA AGCGGAATAT ATCCTGTATC ACATATTCTG 2820

CTGACGCACC GGTGCAGCCT TTTTTCTCCT GCCACATGAA GCACTTCACT GACACCCTCA 2880

TCAGTGCCAA CATAGTAAGC CAGTATACAC TCCGCTAGCG CGCATGCCTG CAGGTCGACT 2940

CTAGAGGATC CTTATTTGCC GACTACCTTG GTGATCTCGC CTTTCACGTA GTGGACAAAT 3000

TCTTCCAACT GATCTGCGCG CGAGGCCAAG CGATCTTCTT CTTGTCCAAG ATAAGCCTGT 3060

CTAGCTTCAA GTATGACGGG CTGATACTGG GCCGGCAGGC GCTCCATTGC CCAGTCGGCA 3120

GCGACATCCT TCGGCGCGAT TTTGCCGGTT ACTGCGCTGT ACCAAATGCG GGACAACGTA 3180

AGCACTACAT TTCGCTCATC GCCAGCCCAG TCGGGCGGCG AGTTCCATAG CGTTAAGGTT 3240

TCATTTAGCG CCTCAAATAG ATCCTGTTCA GGAACCGGAT CAAAGAGTTC CTCCGCCGCT 3300

GGACCTACCA AGGCAACGCT ATGTTCTCTT GCTTTTGTCA GCAAGATAGC CAGATCAATG 3360

TCGATCGTGG CTGGCTCGAA GATACCTGCA AGAATGTCAT TGCGCTGCCA TTCTCCAAAT 3420

TGCAGTTCGC GCTTAGCTGG ATAACGCCAC GGAATGATGT CGTCGTGCAC AACAATGGTG 3480

ACTTCTACAG CGCGGAGAAT CTCGCTCTCT CCAGGGGAAG CCGAAGTTTC CAAAAGGTCG 3540

TTGATCAAAG CTCGCCGCGT TGTTTCATCA AGCCTTACGG TCACCGTAAC CAGCAAATCA 3600

ATATCACTGT GTGGCTTCAG GCCGCCATCC ACTGCGGAGC CGTACAAATG TACGGCCAGC 3660

AACGTCGGTT CGAGATGGCG CTCGATGACG CCAACTACCT CTGATAGTTG AGTCGATACT 3720

TCGGCGATCA CCGCTTCCCT CATATGCTCT CCTTCGCCTC GCTGGCTCTG CTGCGCGGCA 3780

AGAGGCCCGA AGCTCCCTGC ACCATATTGA GAATCATTCT CAATGTCAAG GGCCCTCGGT 3840

CTCCATGCGA GCTCGAATTC GATATCAAGC TTATCGATAC CGTCGACCTC GAGGGGGGGC 3900

CCGGTACC

**Supplementary Figure 10.** Nucleotide sequence of pGRC5.

Red, region of low %G+C (35%), putative pDEGR-3 origin of replication; light blue, pDEGR-3 *rep*; yellow, p15A ori; light green, *D. radiodurans* catalase gene promoter (*kat*p); pink, streptomycin/spectinomycin resistance gene (*aad*).

CCCAAATCCT GACCGTGCCA AAGCCCCAGC AAAAACAGGA ATTGGGTGCC GATTTTCTGC 60

GGGTGCTGGG GTGTTACGCA AGATTAGCAC TAAAGTGCAT CTTGCCTCTG AAACTGCGTT 120

CCAGAGGGGA AAAAACCAGG GTGCTGTTAA TTTTAATTAC TGGTCAACTT TTGGGCCGTT 180

TTTCGAGGGG TCAATGCGCG AGAAATGGCC CAAAAAATCA GCAGGGAGGG AGGTGCTGGA 240

CCTGCCGTTC ACCCCAACTG GCAACACCCC GCTGGGAGGC GGGGTGCTAA GGTAATTCTC 300

GAAGCCAGTC AAAAGTGTCT CCAAACACCG TTGATTGAGC CATCCCACTC CGAAGGACCA 360

CTTCCGATGA ACAATCAAAA GCAGGACAGC TATACCGATA GGGTACGGCA AATCGCCCCC 420

GTAGTTCCAC TCCTGGACCT CATTGTTAAG CCCCTCATCC CTGAACGAGC AGCCCCTAAT 480

CCCTATCTGG AGATGGGCCT AGCCGCCCTG CGTGGCGAGC CGGTGGACAC CCGCCGCGCC 540

CAGGTGCAGC CCAGCAAGCA GTTCCAGAAG GCCAGCCGCC CAGCCCCAGC CGCCCCCCCC 600

CTTGTGCTGA CCTCAGACCA GGGCGAGGAC CAGGAGGCCG CCATGCTGCT GCCCGAGATC 660

GTCGAAGAGA CGCGCCGCCG CCTGGAGAAG CAGGCCCGCG AGAACCGCGA GACGCGCCGC 720

GCCGAACAGC GCGCCCGCTT TCACCAGCTC GTGACCGCCC CCACCGACGA GGCCGCGCCG 780

GACTTCGTGC CGCCTGTGAG CCTTCAGGAA GGCCGTCAGC CCCCCAGCGG CAGTGTGCGC 840

GTCACCTTGC CCCGCCCTGA AGTCCCTGGG CCTGCCTGGG GCGAGCTGAG CGCCGACGCG 900

GTGCTTGATT CGGTGCCGTG GCTGAGCAAG GGCGCGCGGC TGGTGTTCCG CATCATGCAC 960

ATGCTGGCTG TGGCGAAGGC CCAGGACTGC CGGTATCCGG TGATCCCCAA CTCGGCGGCC 1020

TTCCACACGC CCCAACTGTT GCTGGCCTTC GTGGCGCGGT ACACCACCCG GCACTTCCGG 1080

CGACTGACCA ATGAGCTGGA GCACGCTGGG GTGATTGACG GCGGCGCGCA CGCCGCGAAG 1140

GTCAAGACCG GCATCGGCAC CACCCGGCAC CTGTGGGACG GCTCGATCTG GGCGGTCAAG 1200

CTACGGCCCA GCACCTGTGA CGCCTACCTG TCCCCCGAGG ACTGGCAGCA CGAGTGGCGA 1260

GACTTCCAGG CGGACTTGGA GTCGGGCAGG ACGGCGAAAA AGCTAATGTC CTATCTAAAC 1320

ACCTGTGAGG GTTTAGCCAG GCAGGAGCAC GTCCTGAAAA CCTGGGCCGT TAATCCCAAT 1380

GCGAAAATGA CTTCGTTGTG TTTAGAGCGG ACATTTCAGG CCGAGCAGAA AATGACCCTC 1440

CAGGACACGG TTTATGCCCT GCCGCTGATC GGGGAACTGA GCGAACTCAA GCAGGCCGGA 1500

GCCATCGGAA ACGCGGCGTC GATCATCTCG CACGCCCTGG GCGACAGCCA CAGCCGAAGA 1560

TTCTGGTGTG GGTTGCTGTG GGCAGCCACC CGGAACGGCT CTTTAGAGGC GTTCTCGGCC 1620

CAGTTGCTGC GGTTGCTGGC GGATGTGCAG GAGTCCAGCG AGCTGAGAAA CCCCGGCGCG 1680

CTGTTCGCGG CCCGCCTTCG CAGCGCCTGA CCTTCGGGGA CCGCGCCCGA AGACCAGGCG 1740

CTGCTCAGTT GGGCTTGAGG GTGATTACGT TCTGAGCATA TGACCGCATG AGGACATCTG 1800

TTAATAGCGT AAAAATCTCT CTGCTCTAAT GACATTTAGG GCAGAGAAAC TATAGAAAAC 1860

TTATTCGGTT TTTCTTGCAG AAGAGATTAG AACTAGTAAT CAGTTCTATT TTTTTATTAA 1920

ACAGATTTAT TCTCTCAACT ATTATAATTC CTATACCGAA GAGTACGAAG ATGGATGCCA 1980

AGAGCATAGT CATTACTCCC TTTTGCATTG TCGCTCCTTT CAAATCGTAT GATACAGTGT 2040

TCTGACGCGG GTAAAATTTT GGTTTCACCA CTCACGCACT TTTGGAGGCC CGATCATGTC 2100

TGAGGGTACC GAGCTCGAAT TCACTGGCCG TCGTTTTACA ACGTCGTGAC TGGGAAAACC 2160

CTGGCGTTAC CCAACTTAAT CGCCTTGCAG CACATCCCCC TTTCGCCAGC TGGCGTAATA 2220

GCGAAGAGGC CCGCACCGAT CGCCCTTCCC AACAGTTGCG CAGCCTGAAT GGCGAATGGC 2280

CATGACCAAA ATCCCTTAAC GTGAGTTTTC GTTCCACTGA GCGTCAGACC CCGTAGAAAA 2340

GATCAAAGGA TCTTCTTGAG ATCCTTTTTT TCTGCGCGTA ATCTGCTGCT TGCAAACAAA 2400

AAAACCACCG CTACCAGCGG TGGTTTGTTT GCCGGATCAA GAGCTACCAA CTCTTTTTCC 2460

GAAGGTAACT GGCTTCAGCA GAGCGCAGAT ACCAAATACT GTTCTTCTAG TGTAGCCGTA 2520

GTTAGGCCAC CACTTCAAGA ACTCTGTAGC ACCGCCTACA TACCTCGCTC TGCTAATCCT 2580

GTTACCAGTG GCTGCTGCCA GTGGCGATAA GTCGTGTCTT ACCGGGTTGG ACTCAAGACG 2640

ATAGTTACCG GATAAGGCGC AGCGGTCGGG CTGAACGGGG GGTTCGTGCA CACAGCCCAG 2700

CTTGGAGCGA ACGACCTACA CCGAACTGAG ATACCTACAG CGTGAGCTAT GAGAAAGCGC 2760

CACGCTTCCC GAAGGGAGAA AGGCGGACAG GTATCCGGTA AGCGGCAGGG TCGGAACAGG 2820

AGAGCGCACG AGGGAGCTTC CAGGGGGAAA CGCCTGGTAT CTTTATAGTC CTGTCGGGTT 2880

TCGCCACCTC TGACTTGAGC GTCGATTTTT GTGATGCTCG TCAGGGGGGC GGAGCCTATG 2940

GAAAAACGCC AGCAACGCGG CCTTTTTACG GTTCCTGGCC TTTTGCTGGC CTTTTGCTCA 3000

CATGTTCTTT CCTGCGTTAT CCCCTGATTC TGTGGATAAC CGTATTACCG CCTTTGAGTG 3060

AGCTGATACC GCTCGCCGCA GCCGAACGAC CGAGCGCAGC GAGTCAGTGA GCGAGGAAGC 3120

GGAAGAGCGC CCAATACGCA AACCGCCTCT CCCCGCGCGT TGGCCGATTC ATTAATGCAG 3180

CTGGCACGAC AGGTTTCCCG ACTGGAAAGC GGGCAGTGAG CGCAACGCAA TTAATGTGAG 3240

TTAGCTCACT CATTAGGCAC CCCAGGCTTT ACACTTTATG CTTCCGGCTC GTATGTTGTG 3300

TGGAATTGTG AGCGGATAAC AATTTCACAC AGGAAACAGC TATGACCATG ATTACGCCAA 3360

GCTTGCATGC CTGCAGGTCG ACTCTAGAGG ATCCGTGTTT CAGTTAGCCT CCCCCATCTC 3420

CCGATCCGGA CGAGTGCTGG GGCGTCGGTT TCCACTATCG GCGAGTACTT CTACACAGCC 3480

ATCGGTCCAG ACGGCCGCGC TTCTGCGGGC GATTTGTGTA CGCCCGACAG TCCCGGCTCC 3540

GGATCGGACG ATTGCGTCGC ATCGACCCTG CGCCCAAGCT GCATCATCGA AATTGCCGTC 3600

CACCAAGCTC TGATAGAGTT GGTCAAGACC AATGCGGAGC ATATACGCCC GGAGGCGCGG 3660

CGATCCTGCA AGCTCCGGAT GCCTCCGCTC GAAGTAGCGC GTCTGCTGCT CCATACAAGC 3720

CAACCACGGC CTCCAGAAGA AGATGTTGGC GACCTCGTAT TGGGAATCCC CGAACATCGC 3780

CTCGCTCCAG TCAATGACCG CTGTTATGCG GCCATTGTCC GTCAGGACAT TGTTGGAGCC 3840

GAAATCCGCG TGCACGAGGT GCCGGACTTC GGGGCAGTCC TCGGCCCAAA GCATCAGCTC 3900

ATCGAGAGCC TGCGCGACGG ACGCACTGAC GGTGTCGTCC ATCACAGTTT GCCAGTGATA 3960

CACATGGGGA TCAGCAATCG CGCAGATGAA ATCACGCCAT GTAGTGTATT GACCGATTCC 4020

TTGCGGTCCG AATGGGCCGA ACCCGCTCGT CTGGCTAAGA TCGGCCGCAG CGATCGCATC 4080

CATGGCCTCC GCGACCGGCT GCAGAACAGC GGGCAGTTCG GTTTCAGGCA GGTCTTGCAA 4140

CGTGACACCC TGTGCACGGC GGGAGATGCA ATAGGTCAGG CTCTCGCTGA ATTCCCCAAT 4200

GTCAAGCACT TCCGGAATCG GGAGCGCGGC CGATGCAAAG TGCCGATAAA CATAACGATC 4260

TTTGTAGAAA CCATCGGCGC AGCTATTTAC CCGCAGGACA TATCCACGCC CTCCTACATC 4320

GAAGCTGAAA GCACGAGATT CTTCGCCCTC CGAGAGCTGC ATCAGGTCGG AGACGCTGTC 4380

GAACTTTTCG ATCAGAAACT TCTCGACAGA CGTCGCGGTG AGTTCAGGCT TTTTCATATG 4440

CTCTCCTTCG CCTCGCTGGC TCTGCTGCGC GGCAAGAGGC CCGAAGCTCC CTGCACCATA 4500

TTGAGAATCA TTCTCAATGT CAAGGGCCCT CGGTCTCCAT GCGAGCTCGA ATTCGATATC 4560

AAGCTTATCG ATACCGTCGA CCTCGAGGGG GGGCCCGGTA C 4601

**Supplementary Figure 11.** Nucleotide sequence of pZT29H.

Pink, pUE30 *rep*; red, region of low %G+C (35%), putative pUE30 origin of replication; yellow, pUC19 origin of replication; light green, *D. radiodurans* catalase gene promoter (*kat*p); gray, hygromycin resistance gene (*hyg*).

CTCGAGCCAG TTCTCGCGTG TCAGCCGAAC TTTTTCAAGC CCTGCCGGAA ACTGTCGGGT 60

ACTGAGGCTC ATGGGTATGC AGAAAAGACC CAAGGCCGTG TGAAGCCCCG GAGTGCTGGG 120

AGAGGCTTAC CCTGCTTGCT GAACCTGACT GGTCAGGGAG GAGGACGGAT GCGATTCGTG 180

GTGGCCTTTG ACGGAAGTGA GCCCAGCCAG CGCGCCTTGA AGCAGGCCAT AACGCTCGGG 240

AAGGCGGCAG GCGCCGAGCT GGAGGTCGTG ACTGTGATCG AGGAGCCGCC GGTCAGCGGC 300

TTCGTTCAGT GGGCGGGACT GAATCCCGAC TACGTCCGGG AGATGCTGGA GCAGAATGCC 360

CGGCAGGCGC AGCAGACGGC GCAGACACTC ATCGGGGAGG CGGGCGTGCA GGGGAGGGTT 420

CAGACCCTGA GGGGCCGTCC AATCGAGGTG CTCACCGAGG CGGCGCACGG CGTGGACCTG 480

CTGGTGGTCG GCACCCATGG GTACCTGCGC GGTGGCGTCT TCGGCGCTCA CGCTGGCAAA 540

CGGCAGCGCC GCCGAGTTCG GCACGGGGGC CGCCCCGCTG GTCGTGTGCG AGGCGGGGAC 600

GGTGTACGCG CCGACGAACA CGCCCGCGCC TGCCACGCTG GAACTGTCCG ACATGGTGCA 660

GTCGTCCACC TTCACCACGA GCGCCGGGCG GTTCGGGGCG CTGGTGAGCT TCGCGCAGGT 720

GCCCGATAAG GCGGTGATGC TGTGGACGAA GTATCAGTAT CAGCAGGCCG AGGAATGTGG 780

AGCCGGAGAC GCCTATGCGG TGGGCGCGTC CGGCGAGTGG CAGACCCTCA GCAGCCCGGC 840

GAACACCCGC CGTTTCTTTG TCGCGGTGGA TGAGCAAAAG ATGGTGCAGG AGCATTACGG 900

CCTGGCCTAC CAGTTCGCCT TCAAGGACGA AGGGACACCA CACGGTGAAA CCCTGCTCAG 960

GGAAGGGCTT CTGTGCTTCC CTGGTGCGAC CCCCAGCTAA CAACGCCGAG CCATTGGCCC 1020

GCCGCCTGTT TCCCCTCGGG CGGCGGGCCT TTGCGTGACA AATCGCCTCT GTTCTGCTTT 1080

ACTAGGCCAA ATGACTAAAC CCCCACCTTT CGGTGAGGGC TTTGTCAGAG TGCAGGGTCA 1140

CAAACCCCAG CTATCTCGTC GCTGAGAGCA TGGTAGACCG GGACCCTCCC CTCTGACAAG 1200

AGGGGAGGCT TTTTTCTTGA GACACAATCC ATTCATGGTC CGACTCCTCG AAGCGGGCCT 1260

GATTTCGCAG ATTTCACTGC AGGAAGGTAA CCATGCGCCT AAACCTGCGC TCACACGGGA 1320

AATTTCCAGC TCGGCCCGGT CCATCTCGCC GCCGCCCGTG CCCACCATCG CCCAGCAGTC 1380

CCAGCCCGCG CCCGCGCCGT TGACCATCGC CACACGCGCC GAACTGGTGC AGACCCTCAC 1440

CGCCTGCCTA GATGACGCCC CCTGCCGCGA GTCGGCCCGG CGCATCTTCC GCGCCCTGTT 1500

CTCGGTGGCG CTGGACGTGG CCCGCGCCTG CGGCTATGCG GAACACGTCA CCCGCGCTGT 1560

GTTCCACCTG CCCGCCGAGC TGCTCATGGT GCATGTGGGT CTGAAGAAGT CCGCCTTCTA 1620

CGAGAACCTT CAATATCTGC GCCGGGTCGG GCTGGTGGCC TGTGATGCCC ACATGGGCGA 1680

CCTGCGCGGC GAGTCTGTGG CAACCGGGAC GCTGTGGGCC GTGACGCTCA AGCCGAGGCG 1740

CGTGCTGGAA GGGAAGGCGG GATATGTCCG CCTGATGCAT GACGATTGGG GCCGGAACTG 1800

GCGCGACCTG AACGCCGACG CGAAGGCCGG GCGCACCGTC TACAACATGC TCCACCCTCT 1860

CCCTAAGAGC GTGCCGGAGA GTCAAGAACC CTGTGAGGAT GTGACAGGCG GGGAACTGCG 1920

CGAAAAAAGC GCCCCGGTTG CCGTGACATA CGAAGTTGTG AGCACCTGGG CGATAAAATC 1980

CGCTCTTCCC TCCCCCTCCG ATAACATGAC TGTCCGGCCC GCGCCCTCCC TCGCTGAAAA 2040

CGTCGTGTGG GAGCTTGCCG ACGCCCACCG GGAAACCCGC CCGGCCTACC GCGCCGAGAT 2100

CGTGGACAGG CAGGCGCGGG CGCTAGCGGC AGCTTTCGGG GACGGGGCCG ACTCGCTGGG 2160

CTTCTGGCGG AAACTCATTT GGAACATCAC CCGCGCCGCC GACGCTGGCC GGGACGTGTC 2220

CGACGACGTG GGCGCGGTGC TGGTGCGCGT GCTGCGCGAC GTGAAGCACG ACCAGACCGC 2280

AGGCGGAACG CCGCCCCGCA ACCTCGCCGC CGTGGTCAAT GCCGCGCTGG CCGACCTGCT 2340

GGGCAGGCTG CGTGAGTACG AAGGCCAGCG TGTGGGGTCA GGCCCGACCA GGCGCAGTTC 2400

GCGGCCTGAA AAAACAGGGT CACAGAAAGG CCGAGAAGGG GCCTGAAAAG AAGTCCGAGG 2460

GATAAAAGCC CCCGGAAATG CGTCATGTGC AAATTTACGT CCTGATATGA TTCACGGAAA 2520

TATGACCCGA ACGTTTCTTA ATACGTTTAT AGATAGTTTT TTAGTTCGCT GGGCTAACTA 2580

TTTCGGCTTG GTTCTCCTGG CGGTTGGTCT GGCGAAAGAT TTTCAATGGG ATGTGGCCGC 2640

TGCTGCTGTG GCTCTTTTCG TGGCGTTGTT GGCACAAGAA TGGCGGCTTT ATTATAAAAT 2700

CAACGCGCCA AAAACTTATC TGAAACATGA CAGAAAAATT TTTATCGATT TGGTTAACCG 2760

GTCCCCATCA ATGGGAAGTA TTGAATTTCT GAGAAGCCAT GACTTTAGTG ATACTTTCCA 2820

TATAAGTGAA CTGGATCAGT TAGACAGTTT TAATATGTCA AGCAACGATA AGAGTGAAGA 2880

ATTTTTAGAT ATTGAAATAG AGAAGCGACG TGAGACACTT GTGCGTTCCT GTCTGCAAAT 2940

GTCCCATTTG ATTGGACTAA AGACATACCC TAACGGCTCA GGAGGACTAC AAGGCGTTCC 3000

TTCGGAATGG TCGGAGAGTT GGCGTTACAA GGATGATCCA GAATTGGGTT CTCAGTTTGC 3060

TGATAGATAC AAAGAACACG TCAAGACCCT GAACGCGGCT GCAAGAATGG TAGCTGAGGC 3120

GTACAGCTTG CATGCCTGCA GGTCGAATCG GATATCGAAT TCGAGCTCGC ATGGAGACCG 3180

AGGGCCCTTG ACATTGAGAA TGATTCTCAA TATGGTGCAG GGAGCTTCGG GCCTCTTGCC 3240

GCGCAGCAGA GCCAGCGAGG CGAAGGAGAG CATATGGAGA AAAAAATCAC TGGATATACC 3300

ACCGTTGATA TATCCCAATG GCATCGTAAA GAACATTTTG AGGCATTTCA GTCAGTTGCT 3360

CAATGTACCT ATAACCAGAC CGTTCAGCTG GATATTACGG CCTTTTTAAA GACCGTAAAG 3420

AAAAATAAGC ACAAGTTTTA TCCGGCCTTT ATTCACATTC TTGCCCGCCT GATGAATGCT 3480

CATCCGGAAT TCCGTATGGC AATGAAAGAC GGTGAGCTGG TGATATGGGA TAGTGTTCAC 3540

CCTTGTTACA CCGTTTTCCA TGAGCAAACT GAAACGTTTT CATCGCTCTG GAGTGAATAC 3600

CACGACGATT TCCGGCAGTT TCTACACATA TATTCGCAAG ATGTGGCGTG TTACGGTGAA 3660

AACCTGGCCT ATTTCCCTAA AGGGTTTATT GAGAATATGT TTTTCGTCTC AGCCAATCCC 3720

TGGGTGAGTT TCACCAGTTT TGATTTAAAC GTGGCCAATA TGGACAACTT CTTCGCCCCC 3780

GTTTTCACGA TGGGCAAATA TTATACGCAA GGCGACAAGG TGCTGATGCC GCTGGCGATT 3840

CAGGTTCATC ATGCCGTTTG TGATGGCTTC CATGTCGGCA GAATGCTTAA TGAATTACAA 3900

CAGTACTGCG ATGAGTGGCA GGGCGGGGCG TAATTTTTTT AAGGCAGTTA TTGGTGCCCT 3960

TAAACGCCTG GTTGCTACGC CTGAATAAGT GATAATAAGC GGATGAATGG CAGAAATTCG 4020

TCGAAGCTCT AGAGGATCCT CTAGAGTCGA CCTGCAGGCA TGCAAGCTTG ACTCTAGAGG 4080

ATCCCCGGGT ACCGAGCTCG AATTCACTGG CCGTCGTTTT ACAACGTCGT GACTGGGAAA 4140

ACCCTGGCGT TACCCAACTT AATCGCCTTG CAGCACATCC CCCTTTCGCC AGCTGGCGTA 4200

ATAGCGAAGA GGCCCGCACC GATCGCCCTT CCCAACAGTT GCGCAGCCTG AATGGCGAAT 4260

CAATTCCCGA CAGTAAGACG GGTAAGCCTG TTGATGATAC CGCTGCCTTA CTGGGTGCAT 4320

TAGCCAGTCT GAATGACCTG TCACGGGATA ATCCGAAGTG GTCAGACTGG AAAATCAGAG 4380

GGCAGGAACT GCTGAACAGC AAAAAGTCAG ATAGCACCAC ATAGCAGACC CGCCATAAAA 4440

CGCCCTGAGA AGCCCGTGAC GGGCTTTTCT TGTATTATGG GTAGTTTCCT TGCATGAATC 4500

CATAAAAGGC GCCTGTAGTG CCATTTACCC CCATTCACTG CCAGAGCCGT GAGCGCAGCG 4560

AACTGAATGT CACGAAAAAG ACAGCGACTC AGGTGCCTGA TGGTCGGAGA CAAAAGGAAT 4620

ATTCAGCGAT TTGCCCGAGC TTGCGAGGGT GCTACTTAAG CCTTTAGGGT TTTAAGGTCT 4680

GTTTTGTAGA GGAGCAAACA GCGTTTGCGA CATCCTTTTG TAATACTGCG GAACTGACTA 4740

AAGTAGTGAG TTATACACAG GGCTGGGATC TATTCTTTTT ATCTTTTTTT ATTCTTTCTT 4800

TATTCTATAA ATTATAACCA CTTGAATATA AACAAAAAAA ACACACAAAG GTCTAGCGGA 4860

ATTTACAGAG GGTCTAGCAG AATTTACAAG TTTTCCAGCA AAGGTCTAGC AGAATTTACA 4920

GATACCCACA ACTCAAAGGA AAAGGACTAG TAATTATCAT TGACTAGCCC ATCTCAATTG 4980

GTATAGTGAT TAAAATCACC TAGACCAATT GAGATGTATG TCTGAATTAG TTGTTTTCAA 5040

AGCAAATGAA CTAGCGATTA GTCGCTATGA CTTAACGGAG CATGAAACCA AGCTAATTTT 5100

ATGCTGTGTG GCACTACTCA ACCCCACGAT TGAAAACCCT ACAAGGAAAG AACGGACGGT 5160

ATCGTTCACT TATAACCAAT ACGCTCAGAT GATGAACATC AGTAGGGAAA ATGCTTATGG 5220

TGTATTAGCT AAAGCAACCA GAGAGCTGAT GACGAGAACT GTGGAAATCA GGAATCCTTT 5280

GGTTAAAGGC TTTAAGATTT TCCAGTGGAC AAACTATGCC AAGTTCTCAA GCGAAAAATT 5340

AGAATTAGTT TTTAGTGAAG AGATATTGCC TTATCTTTTC CAGTTAAAAA AATTCATAAA 5400

ATATAATCTG GAACATGTTA AGTCTTTTGA AAACAAATAC TCTATGAGGA TTTATGAGTG 5460

GTTATTAAAA GAACTAACAC AAAAGAAAAC TCACAAGGCA AATATAGAGA TTAGCCTTGA 5520

TGAATTTAAG TTCATGTTAA TGCTTGAAAA TAACTACCAT GAGTTTAAAA GGCTTAACCA 5580

ATGGGTTTTG AAACCAATAA GTAAAGATTT AAACACTTAC AGCAATATGA AATTGGTGGT 5640

TGATAGCGAG GGCCGCCCGA CTGATACGTT GATTTTCCAA TTTGAACTAG ATAGACAAAT 5700

GGATCTCGTA ACCGAACTTG AAAACAACCA GATAAAAATG AATGGTGACA AAATACCAAC 5760

AACCATTACA TCAGATTCCT ACCTACATAA CGGACTAAGA AAAACACTAC ACGATGCTTT 5820

AACTGCAAAA ATTCAGCTCA CCAGTTTTGA GGCAAAATTT TTGAGTGACA TGCAAAGTAA 5880

GTATGATCTC AATGGTTCGT TCTCATGGCT CACGAAAAAA CAACGAACAC CACTAGAAAC 5940

AATACTGGCA AAATACGGAA GAATCTGAGG TTCTTATGGC TCTTGTATCT ATCAGTGAAG 6000

CATCAAGACT AACAAACAAA AGTAGAACAA CTGTTCACCG TTACAT 6046

**Supplementary Figure 12.** Nucleotide sequence of pRADN8.

Yellow. pUE10 *rep*; gray, region of low %G+C (32%), putative pUE10 origin of replication; light green, *D. radiodurans* catalase gene promoter (*kat*p); red, chloramphenicol resistance gene (*cat*); light blue, pSC101 origin of replication; turquoise, pSC101 *rep*.


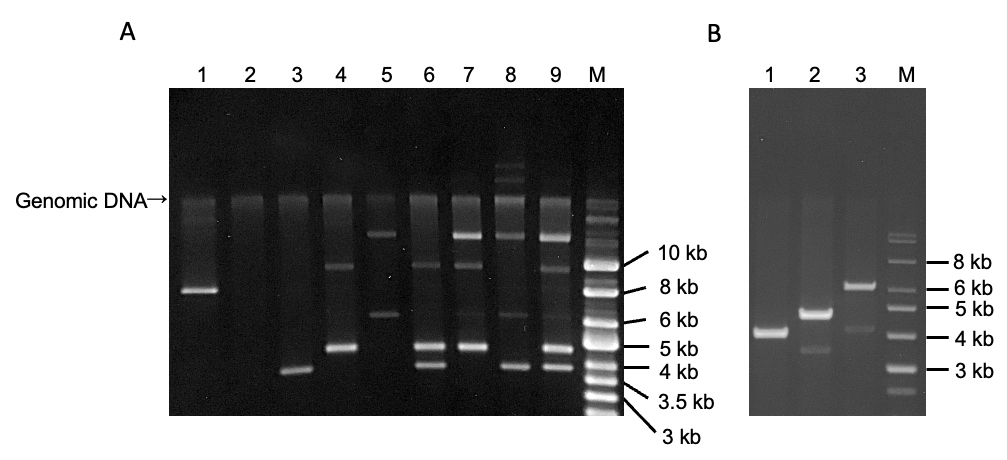


**Supplementary Figure 13.** Plasmid profiles in *D. grandis* transformants

(**A**) Lane 1, the *D. grandis* wild-type strain carrying pDEGR-3 but no shuttle vector; lane 2, the *D. grandis* strain TY3 without a shuttle vector; lane 3, strain TY3 carrying pGRC5; lane 4, strain TY3 carrying pZT29H; lane 5, strain TY3 carrying pRADN8; lane 6, strain TY3 carrying pGRC5 and pZT29H; lane 7, strain TY3 carrying pZT29H and pRADN8; lane 8, strain TY3 carrying pGRC5 and pRADN8; lane 9, strain TY3 carrying pGRC5, pZT29H, and pRADN8. The letter ‘M’ represents a supercoiled DNA ladder manufactured by New England Biolabs (MA, USA). The faint signals in lanes 1–9 are caused by contamination with genomic DNA. In lanes 3, 6, and 9, a band was observed at 3,908 bp, derived from pGRC5; this indicates that pGRC5 is mainly a monomer in *D. grandis*. In lane 4, bands were found at 4601 bp derived from the pZT29H monomer and 9202 bp derived from the pZT29H dimer. Lane 5 shows a band at 6046 bp derived from the pRADN8 monomer, and a band at 12092 bp derived from the pRADN8 dimer. Comparing lanes 3, 4, 6, and 9, no significant differences were observed in plasmid pGRC5 levels present in transformants which harbored pGRC5 alone, and in those that simultaneously contained pZT29H and pRADN8. Similarly, comparing lanes 4, 6, 7, and 9, no significant differences were observed in pZT29H levels between transformants in which pZT29H was present alone and those that simultaneously contained pGRC5 and pRADN8. In contrast, comparing lanes 5, 7, 8, and 9, plasmid pRADN8 was more abundant as a dimer than as a monomer in transformants that simultaneously contained pGRC5 and pZT29H than in transformants that harbored pRADN8 alone. In Lane 8, multiple bands were found at positions less mobile than the genomic DNA, probably because pRADN8 formed more multimers than dimers in transformants carrying pRADN8 and pGRC5. (**B**) Plasmids purified from the *D. grandis* transformants was digested with *Xho*I at 37°C overnight. Lane 1, *Xho*I-digested pGRC5; lane 2, *Xho*I-digested pZT29H; lane 3, *Xho*I-digested pRADN8. The letter ‘M’ represents a 1 kb DNA ladder manufactured by Watson (Hyogo, Japan). The faint signals in lanes 2 and 3 are closed circular DNA left over from *Xho*I digestion.

**
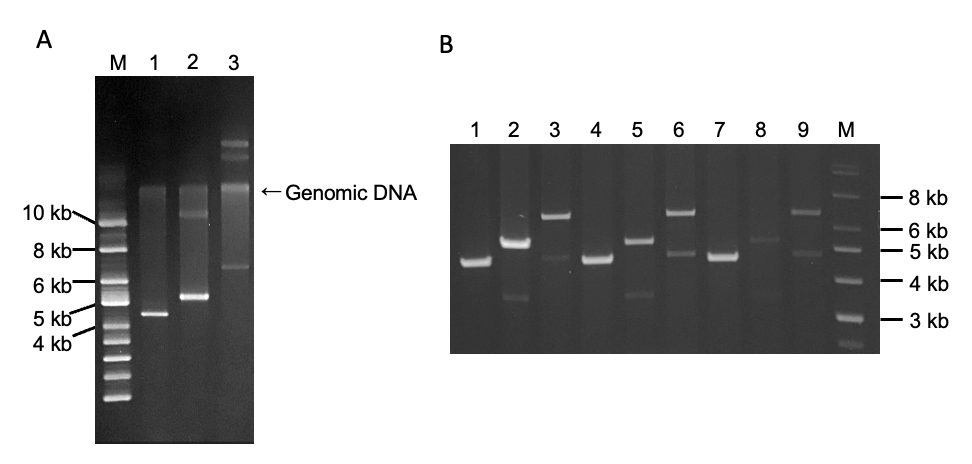
**

**Supplementary Figure 14.** Profiles of different plasmids containing deep sea shrimp luciferase gene (*Nluc*) in *D. grandis* transformants

(**A**) These plasmids were carried by *D. grandis* strain TY3 and *Nluc* was controlled by the *D. radiodurans groES* minimal promoter. The letter ‘M’ represents a supercoiled DNA ladder made by New England Biolabs. Lane 1, strain TY3 carrying pGRC5GN; lane 2, strain TY3 carrying pZT29HGN; lane 3, strain TY3 carrying pRADN8GN. The faint signal in lanes 1–3 is caused by contamination with genomic DNA. In Lane 3, multimers of pRADN8 were observed. (**B**) The *D. grandis* transformants were cultivated in TGY broth without antibiotic addition. After cultivation, plasmids were extracted from the cultures and digested with *Xho*I at 37°C overnight. Each lanes contains plasmid extracts from 8 × 10^8^ colony forming unit of cells. Lane 1, *Xho*I-digested pGRC5GN at the 0 generation; lane 2, *Xho*I-digested pZT29HGN at the 0 generation; lane 3, *Xho*I-digested pRADN8GN at the 0 generation; lane 4, *Xho*I-digested pGRC5GN at the 24 generation; lane 5, *Xho*I-digested pZT29HGN at the 24 generation; lane 6, *Xho*I-digested pRADN8GN at the 24 generation; lane 7, *Xho*I-digested pGRC5GN at the 48 generation; lane 8, *Xho*I-digested pZT29HGN at the 48 generation; lane 9, *Xho*I-digested pRADN8GN at the 48 generation. The letter ‘M’ represents a 1 kb DNA ladder manufactured by Watson.
